# Application of Developmental Regulators for Enhancing Plant Regeneration and Genetic Transformation

**DOI:** 10.3390/plants13091272

**Published:** 2024-05-04

**Authors:** Pingjun Xu, Yinxiao Zhong, Ang Xu, Bingshuang Liu, Yue Zhang, Anqi Zhao, Xiaoming Yang, Meiling Ming, Fuliang Cao, Fangfang Fu

**Affiliations:** State Key Laboratory of Tree Genetics and Breeding, Co-Innovation Center for Sustainable Forestry in Southern China, Nanjing Forestry University, Nanjing 210037, China; pingjun@njfu.edu.cn (P.X.); zhongyinxiao@njfu.edu.cn (Y.Z.); angxa@njfu.edu.cn (A.X.); bsliu@njfu.edu.cn (B.L.); zy1103793748@163.com (Y.Z.); anqi@njfu.edu.cn (A.Z.); xmyang@njfu.edu.cn (X.Y.); mingmeiling@njfu.edu.cn (M.M.); fuliangcaonjfu@163.com (F.C.)

**Keywords:** somatic embryogenesis, plant regeneration, genetic transformation, developmental regulators

## Abstract

Establishing plant regeneration systems and efficient genetic transformation techniques plays a crucial role in plant functional genomics research and the development of new crop varieties. The inefficient methods of transformation and regeneration of recalcitrant species and the genetic dependence of the transformation process remain major obstacles. With the advancement of plant meristematic tissues and somatic embryogenesis research, several key regulatory genes, collectively known as developmental regulators, have been identified. In the field of plant genetic transformation, the application of developmental regulators has recently garnered significant interest. These regulators play important roles in plant growth and development, and when applied in plant genetic transformation, they can effectively enhance the induction and regeneration capabilities of plant meristematic tissues, thus providing important opportunities for improving genetic transformation efficiency. This review focuses on the introduction of several commonly used developmental regulators. By gaining an in-depth understanding of and applying these developmental regulators, it is possible to further enhance the efficiency and success rate of plant genetic transformation, providing strong support for plant breeding and genetic engineering research.

## 1. Introduction

Plant regeneration refers to the process by which plants autonomously repair or replace damaged structures, demonstrating important adaptive capabilities [1]. This ability has been exploited in tissue culture systems for plant propagation, plant genetic transformation, and genome editing [2]. Plant tissue culture regeneration is primarily achieved through organogenesis and somatic embryogenesis pathways. Regeneration induction in the plant genetic transformation system is a crucial step in determining its success. For monocotyledonous plants, regeneration induction mainly relies on somatic embryogenesis; however, for dicotyledonous plants, organogenesis is the primary pathway [3].

Plant genetic transformation is a significant technological advancement in modern science. It promotes a fundamental understanding of plant biology and paves the way for crop improvements and commercial cultivation [4]. With the advancements in genetic engineering and tissue culture techniques, *Agrobacterium*-mediated genetic transformation and tissue culture techniques have helped researchers make significant progress in inducing plant regeneration [5]. However, the establishment of efficient genetic transformation systems is often limited by species and genotypes, as the regenerative capacity of plants varies greatly between different species and even among different varieties therewithin. This further hinders progress in terms of commercializing genetically modified and new, gene-edited crop varieties [2,6]. Regeneration efficiency directly impacts the success rate of targeted gene transformation in the tissue culture process. Therefore, improvements in regeneration and transformation efficiency are crucial for functional gene research and plant transgenic breeding [2,7,8].

In-depth studies of the process of plant somatic embryogenesis have identified the key regulatory genes, which are collectively known as developmental regulators [9]. Research has shown that the overexpression of these developmental regulators in plants can promote somatic embryogenesis or shoot regeneration, thus enhancing the regeneration and transformation efficiency of tissue-cultured plants [9,10,11].

This review briefly describes the molecular basis of plant regeneration and summarizes the progress of developmental regulator research in promoting plant regeneration and improving plant genetic transformation efficiency. It elucidates the applications of development regulators including *BABY BOOM* (*BBM*), *GROWTH-REGULATING FACTOR* (*GRF*), *WUSCHEL* (*WUS*), *LEAFY COTYLEDON1/2* (*LEC1/2*), *SOMATIC EMBRYOGENESIS RECEPTOR-LIKE KINASE* (*SERK)*, and *WOUND*-*INDUCED DEDIFFERENTIATION1* (*WIND1)* in establishing the genetic transformation systems of different plant species [12,13,14]. Additionally, we discuss prospects for the future application of developmental regulators and explore novel approaches for enhancing plant genetic transformation.

## 2. Molecular Basis of Plant Regeneration

The regenerative capacity of plants stems from the totipotency and pluripotency of cells, properties that play a key role in the regeneration of plant tissues [15]. Under stress, wounding, or hormone treatment conditions, isolated cultured plant cells can induce the production of new tissues or organs, demonstrating the ex vivo-induced ability of cellular pluripotency [16]. An in-depth study of the molecular mechanisms of plant regeneration is important for understanding plant biology and agricultural applications.

**Figure 1:**
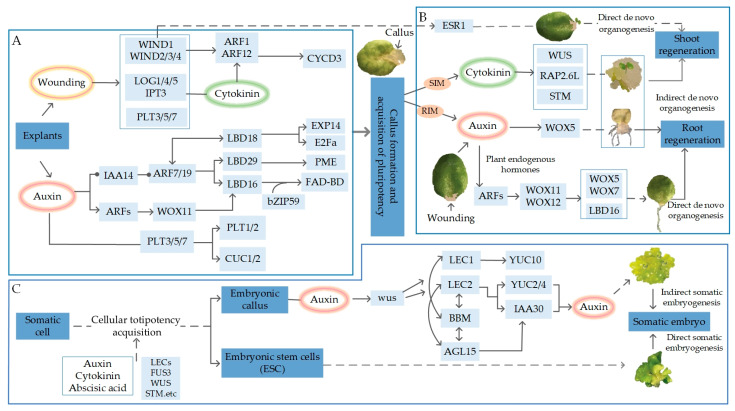
Molecular basis of plant regeneration [19]. (**A**) Molecular basis of callus formation and acquisition of pluripotency. (**B**) Molecular basis of de novo shoot/root organogenesis. (**C**) Molecular basis of somatic embryogenesis. Light-blue boxes indicate genes involved in regulation; arrows and rounded ends indicate activation or repression of gene expression, respectively; solid lines indicate direct regulation; and dotted lines indicate indirect regulation whose mechanisms are not yet clear.

The molecular mechanisms of de novo organogenesis (including root and shoot regeneration) and somatic embryogenesis are explained in Figure 1. A group of rapidly dividing cells, collectively referred to as callus, is produced on the plant at the site of wounding, or on the explant in tissue culture [17]. The non-embryonic callus is seen during de novo organogenesis, which is pluripotent and capable of regenerating roots and shoots; The embryonic callus is seen during somatic embryogenesis, which reflects the totipotency of cells [17,18].

The formation of non-embryonic callus can be induced by applying auxin and cytokinin or in response to wounding and other types of severe stress [17]. Callus developed on callus-inducing medium (CIM) has histological characteristics similar to root meristems [20]. Auxin leads to the degradation of *IAA14* when the explants are cultured on CIM, which subsequently activates *ARF7* and *ARF19*, and in turn, directly activates the expression of *LBD16*, *LBD17*, *LBD18*, and *LBD29* to promote the formation of callus [21]. *LBD* further activates the expression of a range of genes that promote cell proliferation and modulate cell wall properties [22,23]. The *Arabidopsis LBD* transcription factors form a transcriptional complex with the *BASIC REGION/LEU CINE ZIPPER MOTIF 59* (*bZIP59*) transcription factor to co-regulate growth factor-induced callus formation [24]. Furthermore, the *LBD16-bZIP59* complex directly regulates the expression of the cell wall component metabolism gene *FAD-binding Berberine* (*FAD-BD*) [24]. *LBD18* not only regulates the expression of *E2 PROMOTER BINDING FACTOR a* (*E2Fa*), a key transcription factor in the cell cycle, but it also directly regulates the expression of genes that activate cell wall relaxation factors, suggesting that the dynamic remodeling of the cell wall plays an important role in the formation of callus [25]. Wounding activates cytokinin signaling by both inducing biosynthesis and *WIND*-mediated pathways. *ARR*-mediated cytokinin signaling promotes cell cycle re-entry through the activation of *CYCD3*. In addition, wounding also induces *PLT* expression, which is essential for callus formation. Upon incubation on CIM, auxin moves through the *WOX11* and *LBD16*-mediated pathways and pathways involving *PLTs* and *CUC2*, which confers pluripotency to callus cells [26] (Figure 1A).

In the formation of callus from explants, in addition to promoting the proliferation of mid-column sheath-like cells, the characteristics of the cell fate transition to root meristematic tissue are critical for the acquisition of shoot regenerative capacity. In this process, auxin induces the expression of *PLT3*, *PLT5*, and *PLT7*, which in turn activate the expression of the genes *PLT1* and *PLT2*, characteristic of root meristematic tissues, as well as the shoot regeneration characterization factors *CUP-SHAPED COTYLEDON 1* (*CUC1*) and *CUC2*, which confer on the healing tissues the ability to regenerate shoots [27]. It was discovered that the transfer of callus to a shoot-inducing medium (SIM) was requisite for the induction of shoot regeneration [16]. Callus in explants transferred to this medium exhibits broad auxin and cytokinin responses that subsequently become localized to separate domains. *WUS* is eventually expressed in select cells located in the vicinity of the cytokinin response domain. Cytokinin promotes shoot meristem formation via the *ARR*-mediated activation of WUS expression [28]. *PHB*, *PHV*, and *REV* play critical roles in shoot formation through the induction of *WUS*, *STM*, and *RAP2.6L* [29,30,31]. Wounding promotes shoot regeneration via the *WIND1*-dependent activation of *ESR1* [32] (Figure 1B).

The regeneration of roots from leaf explants of *Arabidopsis* is a straightforward and practical method to study root direct de novo regeneration. In this system, without the addition of exogenous hormones to the culture medium, the roots’ de novo generation is entirely dependent on endogenous hormones in the leaf explants. Following leaf excision, the wound rapidly signaled the activation of the auxin pathway genes *YUCCA5/8/9* to trigger the production of auxin [33]. The auxin response is strongly induced near wound sites, where *WOX11* and *WOX12* convert regeneration-competent cells in the vasculature to root founder cells by inducing the expression of *WOX5*, *WOX7*, and *LBD16*. These factors, in turn, direct meristem formation to enhance the regeneration efficiency [34,35] (Figure 1B).

Under specific conditions of environmental stress and/or hormonal induction, somatic cells can either dedifferentiate directly into somatic embryos (direct somatic embryogenesis) or dedifferentiate into embryonic healing tissues and then induce the emergence of somatic embryos on their basis (indirect somatic embryogenesis) [36]. Currently, it has not been determined whether these two modes of development utilize the same mechanism [37]. Upon the transfer of the embryonic callus to an auxin-free medium, the polar transport of growth factor leads to the formation of growth factor maxima near the surface of the callus, during which the cytokinin response domains initially overlap with the growth hormone response domains. Auxin activates the expression of *WUS*, which is required for the activation of the embryonic regulators *LEC1* and *LEC2* [13]. These factors with BBM and *AGL15* form a highly interconnected transcriptional network that works together to promote somatic embryogenesis through multiple positive feedback loops [38,39]. These embryonic regulators further promote the expression of *YUC*, *TAA1*, and *IAA30* to moderate auxin biosynthesis and signal transmission [37,39] (Figure 1C).

In summary, plant regeneration is a complex biological process involving the synergistic action of multiple hormone-signaling pathways and transcription factors. An in-depth study of these molecular mechanisms can provide a deeper understanding of the basic rules of plant regeneration and the theoretical basis and practical guidance for the application of plant regeneration technology in the fields of agriculture and biotechnology.

## 3. Application of BBM Genes in Improving Plant Regeneration

Boutilier et al. discovered the gene that regulates pollen microspore embryogenesis in *Brassica napus* immature pollen and named it the *BABY BOOM* (*BBM*) gene [40]. Studies have shown that the BBM activates downstream genes, such as *LEAFY COTYLEDON1* (*LEC1*), *ABSCISIC ACID INSENSITIVE3* (*ABI3*), *FUSCA3* (*FUS3*), *AGAMOUS*-*LIKE15* (*AGL15*), and *LEAFY COTYLEDON1* (*LEC2*), to initiate somatic embryogenesis in plant cells [41]. *BABY BOOM* (*BBM*) is a transcription factor belonging to the *AP2*/*ERF* (*APETALA2*/ethylene-responsive factor) gene family, playing multiple roles in plant growth and development [42,43,44]. It serves as a crucial regulator during somatic embryogenesis in plant cells. As one of the key genes of embryogenesis, BBM is a switch that initiates and regulates somatic embryogenesis, which is mainly expressed in developing embryos and seeds and can promote cell proliferation and somatic embryonic morphogenesis and construction (Table 1).

The *BBM* gene is considered a key gene in plant embryo formation [14]. In European larch (*Larix decidua*) somatic embryos, Andrea Rupps identified genes related to embryogenesis and analyzed their transcript accumulation during embryogenesis, revealing an increased expression of *LdBBM* in the later stages of development [50]. Sergio L Florez et al. studied the expression patterns of *TcBBM* during somatic and zygotic embryo development in *Theobroma cacao* and found a high expression of *TcBBM* throughout embryo development and the embryogenic callus, with a higher expression during the somatic embryogenesis stage compared to in zygotic embryos and the non-embryogenic callus [55]. The overexpression of the *TcBBM* gene in *Arabidopsis* and *T. cacao* improved the efficiency of somatic embryogenesis. In *Rosa canina*, *RcBBM* showed specific expression in callus and protocorm-like bodies but not in stems, leaves, and flowers, indicating its tissue-specific expression in embryogenic tissues [53]. The heterologous expression of *RcBBM1* and *RcBBM2* in *Arabidopsis* induced adventitious shoot formation. In *Brassica napus*, ectopically expressed *BBM* (*BnBBM1* and *BnBBM2*) can induce somatic embryogenesis in hormone-deficient culture media, which has significant implications for plant genetic transformation. Furthermore, overexpressing *BBM* genes in Chinese white poplar (*Populus tomentosa* Carr.) promoted the formation of embryogenic callus tissue [59]. Subsequently, some of the embryogenic callus tissues successfully regenerated into transgenic plants. The identified *GmBBM1* in soybeans (*Glycine max*) shares a high homology with *AtBBM*. *GmAIL5* and *GmPLT5* are homologous genes of *AINTEGUMENTA*-*like5* and *PLETHORA2*, respectively. The *GmBBM* gene in soybeans showed similar functions to *AtBBM* and *BnBBM*, as it participates in the formation and development of somatic embryos [49]. These findings highlight the crucial role of *BBM* genes as transcription factors controlling embryo development.

The *BBM* gene also plays a crucial role in promoting cell proliferation and regeneration, contributing to plant transformation. Numerous studies have shown that the overexpression of *BBM* in different plant species can enhance cell proliferation and improve plant regeneration capacities [14,45]. Srinivasan et al. explored the effects of *AtBBM* and *BnBBM* on tobacco (*Nicotiana tabacum*), and found similar results in *Arabidopsis* or *B. napus*, in terms of callus formation, leaf curling, and male sterility [60]. Although spontaneous somatic embryogenesis was not observed in transgenic tobacco, the induction of somatic embryogenesis from the hypocotyls of germinating seedlings occurred when cultured on a medium supplemented with cytokinins, indicating that the heterologous expression of *BBM* in tobacco also activates the cell proliferation pathway. The genetic transformation of pepper (*Capsicum* spp.) is challenging due to its limited abilities in terms of embryo formation. However, *BnBBM* expression in sweet pepper (*Capsicum annuum*) can induce a significant amount of cell regeneration, resulting in the generation of abundant embryogenic structures that are easily differentiated into plantlets [47]. This approach has successfully addressed the difficulties in the genetic transformation of pepper. The co-transformation of *BBM* and *WUS* genes has been successfully applied to effectively improve the regeneration and genetic transformation of recalcitrant varieties of various plants, such as maize (*Zea mays*), rice (*Oryza sativa*), and sorghum (*Sorghum bicolor*) [56,57,58].

The *BBM* gene has also been utilized to promote apogamous reproduction in fern plants. Somatic embryogenesis in angiosperms can be considered a form of asexual reproduction. Fern plants can bypass fertilization and directly produce apogamous structures from the gametophyte, completing asexual reproduction [61]. Culturing the model fern plant *Ceratopteris richardii* on a high-sugar medium has been shown to induce the formation of apogamous structures, and overexpressing the *BnBBM* enhances apogamous reproduction in *C. richardii* under sugar-free culture conditions. This research indicates a certain degree of genetic conservation between apogamous reproduction and somatic embryogenesis. In a recent study by Chen et al., a re-evaluation of *BBM* gene expression and functionality was performed using CRISPR mutants in *Arabidopsis* [46]. Their analysis revealed a much earlier expression of *BBM* during embryogenesis compared to previous studies. *BBM* gene expression was identified during the early stages of embryonic cell division and within the suspensor and endosperm, highlighting its presence in various embryonic tissues. The ectopic expression of BBM in the egg cells of *Brassica napus* and *Solanum Lycopersicon* induced haploid embryogenesis, enabling embryo development without fertilization and, thus, demonstrating the role of *BBM* in the gametophytic apomixis of dicotyledonous plants. These findings provide compelling evidence for the critical role of the *BBM* gene in regulating plant totipotency. Table 1 shows the expression of the *BBM* gene and its functions in some species.

## 4. WOX Gene Expression and In Vitro Plant Regeneration

In 1996, the *WUSCHEL* (*WUS*) transcription factor was first discovered in *Arabidopsis* by Laux et al. It is known to play a crucial role in maintaining the apical meristem [62,63]. Since then, numerous *WUSCHEL*-related transcription factors have been identified in various plants, all of which contain a homologous special-shaped box (HB) domain structure [62,64]. These transcription factors belong to the homeobox superfamily and have been collectively named *WOX* transcription factors [64]. *WOXs* play diverse and important roles in plant development. In *Arabidopsis*, a total of 15 *WOX* members have been found, which are *WUS* and *WOX1-WOX14* [65]. During plant development, the embryonic stage is the core constituent ensuring normal growth [66]. Previous studies have shown that *WOX* transcription factors play key regulatory roles in embryonic development in plants, and the differential expression of *WOX* affects the differentiation fate of cells in the early embryonic stage (Table 2).

During development, the process of somatic embryogenesis exhibits significant similarities to that of zygotic embryogenesis [88]. The developmental transitions involved in both embryogenesis and somatic embryogenesis are closely related to a series of molecules that recognize internal signals and external stimuli [89]. During this time, WOXs play a crucial regulatory role in complex biological processes such as embryonic development and somatic embryogenesis [90,91,92]. In *Arabidopsis*, the expression dynamics of different WOX family members affect region-specific transcription during embryonic development, which is instrumental in revealing the developmental mechanism in the *Arabidopsis* embryonic model [65]. For example, *WOX2* and *WOX8* are co-expressed and asymmetrically divided into egg cells and zygotes; these are restricted to the apical and basal daughter cells of the zygote, respectively. *WOX9* responds to signals from the embryo properly; its expression begins in the basal daughter cells of the fertilized egg and is subsequently transferred to the offspring of the apical daughter cells. Similarly, in conifers, *WOX8/9* and *WOX2* genes have also been identified as being active in the initial stages of embryo development [65]. 

In an investigation of de novo organ regeneration, *WUSCHEL-RELATED HOMEOBOX11* (*WOX11*) was identified as the pivotal gene responsible for auxin response and cell fate transition [93]. The crucial involvement of *Arabidopsis WOX11* has been observed in founder cells, where it initiates the development of new organs during the regeneration processes, such as in adventitious roots from detached leaves, adventitious lateral roots from wounded primary roots, and callus formation in tissue culture [34,94,95].

Long and colleagues conducted a comprehensive study on the expression patterns of WOX genes in the somatic embryogenesis of *Liriodendron hybrids* [77]. They used transgenic techniques to systematically observe the process of somatic embryogenesis in *L. chinense* and paid special attention to the expression pattern of the WOX gene at different developmental stages. The research results show that 10 out of 11 LcWOXs were differentially expressed in somatic embryogenesis. Specifically, the expression of LcWUS was the highest on day 2, after the transfer of embryogenic calli from *L. chinense* to the induction medium. LcWOX1 showed gradually increasing expression during the heart-shaped embryo stage, reaching its peak during the plantlet stage. LcWOX2a showed the highest expression level during the torpedo embryo stage. LcWOX3 displayed a similar expression pattern to LcWOX1, with an increase in expression from the heart-shaped embryo to the torpedo embryo stage, reaching its peak during the cotyledonary embryo stage, but decreasing sharply in the plantlet stage. LcWOX4 exhibited increased expression during the heart-shaped embryo stage and maintained relatively high expression levels during the torpedo and cotyledonary embryo stages. On the other hand, LcWOX9 showed extremely high expression levels in the embryogenic callus tissue, while LcWOX13 expression was almost undetectable during somatic embryogenesis [77]. The expression patterns of the WOX genes in the somatic embryogenesis of *L. chinense* indicate that the LcWOX gene plays an important role in the different stages of somatic embryogenesis and the different tissues of post-embryonic development.

During early embryogenesis in tobacco, *WOX* genes play significant roles, with most of them displaying specific expression patterns in different cell types and different stages of embryogenesis [74]. Through RT-qPCR (quantitative real-time reverse transcription PCR) analyses, it was found that *WOX* genes, particularly *WOX2* and *WOX9*, are essential for early embryo patterning. This study contributes to our understanding of the specific roles of *WOX* genes in key developmental processes during embryogenesis [74]. Recent research has also demonstrated that, in tobacco, the co-expression of *WOX* genes, such as the combination of *WOX2* with *WOX8*, or *WOX2* with *WOX9*, promotes remarkable regeneration from freely suspended cells and leaf segments in the absence of cytokinin [75]. The co-expression of these genes can be beneficial for developing cytokinin-free protocols in various species, including some recalcitrant species.

Chen et al. identified homologous genes of *Arabidopsis*, *WUSCHEL (MtWUS)*, *CLAVATA3* (*MtCLV3*), and *WUSCHEL*-related homeobox gene *WOX5* (*MtWOX5*) in *Medicago truncatula* [78]. Their study showed that *MtWUS* and *MtWOX5* are associated with embryonic development, and RNAi experiments demonstrated that the expression of *MtWUS* is crucial for the production of callus tissue and somatic embryos. It has been reported that the functions of *WUS* and *WOX5* are related to maintaining stem cell growth [96]. Studies suggest that *MtWUS* and *MtWOX5* may play roles in inducing stem cell regeneration, which is critical for somatic embryogenesis and root induction. A comparative analysis of the expression of *WOX* and *PIN* genes in embryo sacs and somatic embryogenesis in *M. truncatula* found that the expression levels of *MtWOX11*-like and *MtPIN10* increased in the embryo sacs and were activated during somatic embryogenesis [80]. In contrast, the expression levels of other *WOX* and *PIN* genes were lower in the embryo sacs and did not show the transcriptional activation associated with somatic embryogenesis. Furthermore, they found that the homolog of *AtWOX9* (*STIMPY*) in *M. truncatula*, *MtWOX9-1*, participates in somatic embryogenesis; by altering the expression levels of various genes associated with embryogenesis, it is possible to induce this process in tissue culture [81]. Tvorogova et al. suggested that the regulatory mechanisms governing the early stages of somatic and zygotic embryogenesis may be the same. Additionally, promoting the expression of *MtWOX9-1* could potentially promote plant biotechnology by improving recalcitrant plants’ transformation and regeneration capabilities. More recently, Kadri et al. explored the impact of overexpressing the heterologous *Arabidopsis WUS* gene, regulated by the jasmonate-responsive vsp1 promoter, on the morphogenetic responses of *M. truncatula* explants. The introduction of *WUS* gene expression in leaf explants led to a notable increase in callogenesis and embryogenesis without growth regulators [72]. Similarly, *WUS* gene expression demonstrated the ability to enhance the embryogenic potential of hairy root fragments. These findings suggest that the overexpression of the *WUS* gene can effectively modulate morphogenetic responses in *M. truncatula* explants, opening up possibilities for further research and applications in the realm of plant tissue culture and regeneration.

The *WUS* gene has been identified as a crucial regulator in plant embryogenesis. For example, the overexpression of *PGA6/WUS* in *Arabidopsis* has been demonstrated to induce the transition from a vegetative to an embryogenic state in various tissues, including leaf petioles, leaves, stems, and roots, without the addition of plant growth regulators in the regeneration medium [67]. The expression of *WUS* in *Coffea canephora* has been found to result in the formation of calli and a significant increase of 400% in terms of somatic embryo production [68]. However, it has been observed that the induction of embryogenesis by *WUS* in *Coffea canephora* requires a critical developmental stage and additional hormonal requirements. The expression of *AtWUS* in a recalcitrant cotton species (*Gossypium hirsutum*) was found to enhance embryogenic callus formation by 47.75%. Additionally, *AtWUS* upregulated the expression of *LEC1/2* and *FUS3* in the embryogenic callus of cotton [71]. These findings highlight the potential of *AtWUS* in promoting in vitro plant regeneration through somatic embryogenesis in cotton, which has traditionally been a challenging process. On the other hand, it was noted that the excessive expression of *WUS* led to the emergence of anomalous structures resembling embryos and that the embryos also exhibited leaf-like structural growth [70]. 

Collectively, these investigations indicate that the *WUS* and *WOX* genes play crucial roles in enhancing the ability of plant cells to undergo somatic embryogenesis, thereby greatly influencing plant biology. It has been suggested that the upregulation of these genes increases the frequency of transformations in *Agrobacterium tumefaciens* and in various plant species, both those commonly used in research and those grown for agricultural purposes [11,12,56,57,97]. In terms of in vitro morphogenesis, the recalcitrant nature of *Capsicum chinense* poses a challenge for researchers. However, a related study tackled this issue by conducting an in vitro transformation of *C. chinense* using *Agrobacterium tumefaciens* co-cultivation [69]. In this approach, a system was employed that expresses the heterologous gene *WUSCHEL*, derived from *Arabidopsis*. The overexpression of the *WUSCHEL* gene was demonstrated to facilitate the transition from a vegetative to an embryogenic state.

William Gordon-Kamm and his team found two genes in maize that greatly affected transformation efficiency: *BBM* and *WUS2* [57]. Higher transformation efficiencies have been achieved in many previously non-transformable maize inbred lines via the overexpression of the *BBM* and *WUS2* genes [56]. Furthermore, their study found that the maize *BBM* and *WUS2* genes also promoted transformation in sorghum embryos, sugarcane callus, and indica rice callus. However, the ectopic expression of *BBM/WUS2* affects the growth of regenerated plants, often leading to thickened and distorted phenotypes, as well as developmental abnormalities and sterility. To solve this problem, the researchers conducted a thorough examination and proposed solutions.

The scientists employed the *PLTP* promoter from the phospholipid transfer protein gene and the *Axig1* inducible promoter from the *Axig1* gene to drive the expression of the *BBM* and *WUS2* genes, respectively [58]. This groundbreaking approach made efficient and rapid genetic transformation in maize possible without relying on specific maize genotypes or undergoing the callus culture stage. Following the transformation of *Zm-PLTPpro*-driven *BBM* and *Zm-Axig1pro*-driven *WUS2*, a substantial number of somatic embryos were rapidly generated. These somatic embryos can be directly transformed and grown into plants without the need for callus induction. Importantly, the transformed plants exhibited normal growth and reproductive processes. Recently, the research team led by Ajith Anand developed a method that utilizes an inducible site-specific recombinase (*Cre*) to excise morphogenesis genes [98]. This approach not only optimizes the expression patterns of *BBM/WUS2* genes, resulting in transformed individuals free of morphogenesis genes and selection markers but also enhances transformation efficiency in maize inbred lines. These novel techniques demonstrate great potential for improving the efficiency and precision of maize transformation while eliminating undesired genetic elements. Their application can significantly advance genetic research and breeding programs in maize. 

*TaWOX5*, an important gene in wheat, exhibits predominant expression in the roots and calli, induced by both auxin and cytokinin. This expression pattern suggests that *TaWOX5* is closely associated with root formation or development. Moreover, it is also implicated in the regulation of plant growth regulators (PGRs) during somatic embryogenesis [97]. In a recent study, the experiments of Wang et al. proved that the overexpression of the *TaWOX5* gene significantly improved the transformation frequency of wheat and *T. monococcum*, triticale, rye, barley, and maize, promoting plant regeneration. They also noted a lessened dependence on genotypes. There were few negative effects on the callus differentiation and root development of regenerated plants, but a recognizable phenotype of relatively wide flag leaves was present, which helped us identify *TaWOX5* transgenic wheat plants [86].

## 5. GRF-GIF Chimera Regulates Regeneration in Histoculture

Researchers discovered a rice gene that responds rapidly to gibberellin (GA) treatment and that is believed to be involved in growth and development regulation [99]. They named it the Growth-Regulating Factor (*GRF*). *GRF* is a highly conserved plant-specific transcription factor that typically contains two conserved domains: WRC (Trp, Arg, and Cys) and QLQ (Gln, Leu, and Gln) [100,101]. GRF interacts with its partner, GRF-Interacting Factor (*GIF*), which contains an SNH domain that interacts with the QLQ domain of *GRF*, forming a protein complex. This complex modulates the expression of downstream target genes, thereby regulating various aspects of plant growth and development [102,103,104]. Studies have shown that, in the tissue culture of many species, both *GRF* and *GRF-GIF* fusion proteins can significantly promote cell proliferation and plant regeneration or interact with other transcription factors to enhance plant growth and development [105,106,107] (Table 3). Additionally, the expression of *GRF* is post-transcriptionally inhibited by *microRNA miR396*, forming the *miR396-GRF/GIF* regulatory system, which plays a critical and unique role in plant growth and development [108,109,110].

The overexpression of *GRF* in monocotyledonous and dicotyledonous plants has been shown to enhance their regeneration and transformation efficiency. Debernardi et al. demonstrated that co-transforming *GRF* with its cofactor *GIF* significantly improves the regeneration and transformation efficiency of monocotyledonous durum wheat (*Triticum turgidum* var. *durum*), common wheat (*Triticum aestivum*), rice, and triticale (*Triticale rimpau*), thus accelerating the transformation process [105]. Compared to the empty vector control, the regeneration frequency of *GRF4-GIF1*-transformed materials significantly increased, indicating that the expression of *GRF4* and *GIF1* proteins effectively promotes plant regeneration. Mutations in the potential miR396 target site within *GRF4* further enhance wheat transformation efficiency. Moreover, wheat transgenic lines containing *GRF4*-*GIF1* can induce green shoots on media containing only auxin, allowing for the selection of transgenic plants without antibiotic markers. *GRF4*-*GIF1* also functions in *citrus* (*Citrus sinensis*), where mutations in the *miR396* target site further enhance the activity of grape (*Vitis vinifera* L.) *GRF4-GIF1* fusion, thereby improving *citrus* regeneration capabilities. The latest study found that the overexpression of the *TaLAX1* gene can significantly improve wheat bud regeneration ability. *TaLAX1* may enhance wheat sprout regeneration ability by activating *TaGRF4*, *TaGIF1*, cytokinin synthesis, and the expression of auxin transport-related genes, thus improving genetic transformation and gene editing efficiency [115].

*GRF4-GIF1* has also shown utility in the gene editing of genetically recalcitrant species. Feng et al. constructed watermelon *(Citrullus lanatus*) *ClGRF4-ClGIF1* expression vectors and transformed them into the wild watermelon variety, “TC”, which increased the transformation efficiency of watermelons to 47.02%, which constitutes a nine-fold increase compared to with the control. Mutations in the *miR396* target site further enhanced the transformation efficiency, overcoming the challenge of genotype dependency in watermelon genetic transformation [107]. Interestingly, they combined *ClGRF4-GIF1* with the *CRISPR/Cas9* genome editing tool and achieved efficient gene editing in watermelons, successfully breeding a diploid seedless watermelon, which holds great significance for watermelon breeding [107]. In the latest study, the regeneration efficiency and shooting frequency were assessed in two lettuce genotypes, Cobham Green and *L. serriola accession* Armenian 999, using *GRF4–GIF1* chimeric transgenes derived from tomatoes (*Solanum lycopersicum*) (*GRF4#8–GIF*1), peppers (*GRF4–GIF1*), citrus (*GRF4–GIF1*), and grapes (*rGRF4–GIF1*). Cobham Green demonstrates a consistent regeneration capability, while *L. serriola accession* Armenian 999 shows variable regeneration propensity [114]. In general, a more consistent regeneration capability in the transformed lines was observed.

Besides transforming *GRF4-GIF1* to increase the regeneration and transformation efficiency of wheat, durum wheat, rice, and citrus, the overexpression of *GRF5* in callus cells can also accelerate shoot formation and significantly improve transformation efficiency in multiple species [111,112]. Kong et al. demonstrated that overexpressing *AtGRF5*, *AtGRF6*, and *AtGRF9* in *Brassica napus* can enhance the proliferation of transgenic callus cells [112]. The overexpression of *AtGRF5* and its homologous genes not only promotes cell proliferation and transgenic shoot formation in soybeans and sunflowers (*Helianthus annuus*) cells but also stimulates stem organ growth in different varieties of *Beta vulgaris*, including some that are challenging to regenerate, ultimately leading to the generation of transgenic plants [112]. Additionally, the overexpression of *AtGRF5* and *ZmGRF5-LIKE* genes can facilitate the transformation of maize protoplasts, resulting in the generation of fully fertile transgenic plants. Utilizing genes encoding developmental regulators, such as *AtGRF5*, the transformation efficiency of watermelons has been significantly improved to around 24.73% using the appropriate *Agrobacterium* strain (GV3101), representing a nearly 40-fold increase compared to traditional methods [112]. Furthermore, the co-expression of *GRF5* has been applied in gene editing in the watermelon cultivar WWl50, whereby the inclusion of *GRF5* in *CRISPR/Cas9*-mediated gene editing vectors successfully generated three PDS mutants in 11 transformed watermelon plants, with the homozygous *pds* mutants exhibiting corresponding albino phenotypes [111]. In another study by Zhang et al., they explored the potential of GRF-GIF fusion constructs in genome editing for Cannabis plants [106]. By identifying the similarity with developmental regulators *OsGRF4* and *AtGIF1*, the researchers cloned the endogenous *CsGRF3-CsGIF1* fusion construct from the Cannabis genome. The study revealed a significant improvement in the regeneration efficiency of transgenic Cannabis plants when using the *GRF-GIF* fusion construct compared to control vectors. Combining the *CsGRF3-CsGIF1* fusion construct with the *CRISPR/Cas9* system resulted in a substantial enhancement in regeneration efficiency and the successful editing of specific target genes in Cannabis plants, leading to the generation of four edited Cannabis seedlings displaying albino phenotypes [106].

The *GRF-GIF-BBM* method reported by Chen et al. is a novel transformation approach aimed at enhancing genetic transformation efficiency in maize [113]. This method combines the use of *GRF-GIF* fusion constructs and the *BABY BOOM* (*BBM*) morphogenic regulator gene. Researchers selected *ZmBBM* and fused it with the wheat *GRF-GIF* construct (*TaGRF4-GIF1*), resulting in improved genome editing efficiency in maize backgrounds, B104 and Hi-II. To implement the *GRF-GIF-BBM* method, a vector called pBUE411-GGB was constructed, and immature maize embryos were transformed using the *Agrobacterium*-mediated delivery of the pBUE411-GGB construct. The results demonstrated a significant increase in transformation efficiency, with no adverse effects observed in terms of the expression of the morphogenic regulators *ZmBBM* and wheat *GRF4-GIF1*. Hence, the GGB system holds valuable potential as a tool for genome editing in challenging plant species [113].

With further research on *GRF-GIF* fusion constructs, we believe there may be additional applications. By exploring and optimizing the expression methods and functional mechanisms of *GRF-GIF* fusion constructs, diversified applications can be developed, making greater contributions to plant genetic improvement and sustainable agriculture.

## 6. The Significance of LEC Genes in Plant Somatic Embryogenesis

*LEAFY COTYLEDON* (*LEC*) was initially discovered in *Arabidopsis* and consists of *LEC1* and *LEC2*, which encode two distinct transcription factors. *LEC1* encodes the HAP3 subunit of the CCAAT-binding factor (CBF), containing three structural domains (A, B, and C). The A and C domains are located at the N- and C-termini, respectively, while the B domain is in the central region and is more conserved than the A and C domains. Research has shown that the B domain is a critical region for determining functionality [116]. *LEC2* was first identified and isolated from *Arabidopsis* T-DNA insertion mutants. An amino acid sequence comparison revealed that the central region of *LEC2* shares 43% homology with *FUS3*, both encoding VP1/ABI3-like B3 family transcription factors [117]. *LEC* transcription factors (*LEC1*, *LEC2*, and *FUS3*) play central regulatory roles in embryogenesis and are critically involved in both embryonic morphogenesis and the maturation stages [118] (Table 4). They determine the fate of embryonic suspensor cells and regulate cotyledon characteristics during early embryonic development [117,119,120,121,122]. Additionally, the expression of *LECs* in later stages is associated with the accumulation of storage compounds and the acquisition of embryo desiccation tolerance during seed maturation [117].

Studies in the model plant *Arabidopsis* have demonstrated that expression vectors *35S∷AtLEC1* and *35S∷AtLEC2* can promote spontaneous somatic embryogenesis in vegetative organs [117,129]. For instance, the ectopic expression of *LEC1* in *Arabidopsis* leads to abnormal seedlings exhibiting embryonic features such as non-expanded cotyledons, impaired root elongation, and the emergence of embryo-like structures at the shoot apical meristem. This indicates that *LEC1* can induce the conversion of somatic cells into embryonic cells. Researchers, such as Stone et al., introduced *35S∷AtLEC2* into *lec2-1* and *lec2-5* mutant *Arabidopsis* lines and observed enhanced germination and the formation of adventitious embryogenic cells, eventually resulting in stable transgenic lines. This suggests that *AtLEC2* possesses a stronger embryogenic induction capacity. The studies by Stone et al. also indicate that *LEC* may establish a cellular environment in *Arabidopsis* that promotes the coordinated expression of relevant genes, thereby driving embryonic morphogenesis and maturation [117].

Malgorzata et al. investigated the somatic embryogenic capabilities of *lec1*, *lec2*, and *fus3* mutants in *Arabidopsis* [133]. The results revealed a strong inhibition of embryogenic initiation in the *lec* mutants compared to wild-type *Arabidopsis*. The authors observed very low rates of somatic embryo formation in *lec* mutants, ranging from 0.0% to 3.9%. Furthermore, embryogenic initiation was completely suppressed in the double mutants (*lec1 lec2*, *lec1 fus3*, and *lec2 fus3*), as well as in the triple mutant (*fus3 lec1 lec2*). These findings suggest that the formation of somatic embryos induced by auxin requires the expression of *LEC1* and *LEC2* genes.

Overall, these studies shed light on the crucial roles of *LEC* transcription factors in embryogenesis, establishing a foundation for understanding the mechanisms underlying embryonic development and maturation processes in plants.

In addition to *Arabidopsis*, *LEC* genes have been reported to play a role in embryogenesis in various other plant species, including *M. truncatula*, maize, carrots (*Daucus carota*), *Medicago sativa*, and *Theobroma cacao* [150]. To investigate the changes in gene expression during the development of leaf protoplasts into somatic embryos in purple medic (*M. truncatula*) cultured in a medium containing 2,4-D, Domoki et al. analyzed the relative expression levels of selected genes during the 2,4-D treatment of leaves and the process of somatic embryogenesis [143]. The study revealed that ten genes, including *MsLEC1*, exhibited different expression patterns during the induction and differentiation stages of somatic embryogenesis. This suggests that the expression of these genes may be closely associated with developmental transitions, such as differentiation and dedifferentiation, during somatic embryogenesis. 

Zhang et al. used in situ hybridization to investigate the expression pattern of *ZmLEC1* during the process of somatic embryogenesis in *Z. mays* Hi-II genotype [149]. They found that *ZmLEC1* was strongly expressed throughout the somatic embryo, displaying a similar expression pattern to that of *LEC1* during zygotic embryo development in *Arabidopsis*. This result suggests that *ZmLEC1* may play an important regulatory role in maize somatic embryogenesis and demonstrates how the expression of *ZmLEC1* can serve as an effective molecular marker for detecting the early stages of in vitro somatic embryogenesis. Katsumi Y and colleagues identified and isolated a homolog of *AtLEC1*, named *C-LEC1*, in carrots, in order to better understand the role of *LEC1* in regulating embryonic morphogenesis and seed maturation [137]. Their analysis showed that *C-LEC1* was expressed in embryogenic cells, somatic embryos, and early globular embryos in immature seeds, and the expression driven by the *AtLEC1* promoter complemented the defects of the *Arabidopsis LEC1-1* mutant. Their findings revealed that *LEC1* is an important regulatory factor for both zygotic and somatic embryo development [137,149].

In the regulation network of somatic embryogenesis (SE) in upland cotton, *GhLEC1* has been identified as a direct upstream negative regulator [138]. It negatively regulates the expression of the casein kinase I gene *GhCKI* by binding to the cis-element (CTTTTC) in the *GhCKI* promoter region. By inhibiting *GhCKI* transcription, *GhLEC1* inhibits its expression, thus influencing the transition from cell proliferation to differentiation as well as somatic embryo formation during the late stages of SE. The authors suggest that understanding the complex regulatory network of SE, including the roles of *GhLEC1* and *GhCKI*, as well as their interactions with other factors, can provide insights into the mechanisms of somatic embryogenesis in upland cotton. In another culture of sweet peppers, it was reported that *BBM* and *LEC* genes were highly expressed during direct embryogenesis [134]. However, the independent presence of these genes does not explain the differential responses of pepper varieties to direct embryogenesis. Further research is needed to understand the regulation of this process by other genes and their interactions.

Kwong et al. isolated a gene encoding the CCAAT-box binding factor *HAP3* subunit with high homology to *LEC1* in *Arabidopsis*, belonging to the *LEC1-type* within the *HAP3* gene family; they named it *LEC1-LIKE* (*L1L*) [116]. An analysis showed that the *L1L* gene is mainly expressed during embryogenesis and that mutations lead to defects in plant embryo development. Moreover, introducing the *L1L* gene into *Arabidopsis LEC1* mutants restored the defects caused by *LEC1* mutations, such as embryonic development abnormalities and desiccation intolerance. In conclusion, the *L1L* gene is essential for normal embryo development. *HaL1L* is a gene involved in sunflower embryogenesis. Fambrini et al. studied the role of *HaL1L* in somatic embryogenesis using the sunflower cell line variant *H. annuus × H. tuberosus* (EMB-2) [139]. They found that the ectopic proliferation of adventitious embryos on EMB-2 leaves was associated with the accumulation of *HaL1L* mRNA. High levels of *HaL1L* transcripts were detected in the early stages of sunflower embryogenesis, indicating their involvement in these developmental processes; however, their precise function in embryo development remains to be determined. To further investigate the association between *HaL1L* and somatic embryogenesis, the team continued to study the relationship between *HaL1L* expression and auxin accumulation in EMB-2. They found that *HaL1L* expression was upregulated in the epidermal region where somatic embryos formed and that the content of IAA (auxin) increased [140]. They speculated that *HaL1L* might be involved in somatic embryogenesis through its interaction with auxin, although further research is needed to explore this correlation.

In *Citrus sinensis*, an *L1L* gene named *CsLIL* was identified, and its expression levels in different tissues were analyzed [136]. *CsLIL* showed high expression in embryogenic calluses, somatic embryos, and immature seeds. It was found that the ectopic expression of *CsLIL* in vegetative tissues induced the formation of embryo-like structures, indicating that *CsLIL* can promote the transition of cells from the vegetative to the embryogenic phase. A comparative analysis between *CsLIL* expression in newly formed and long-term cultured embryogenic callus tissues revealed a correlation between *CsLIL* expression levels and embryogenic competence. Collectively, the research results indicate that *CsLIL* plays an important role in regulating embryogenesis in citrus [136]. In a recent study, Zheng Liu et al. discovered that the *CsFUS3* gene is preferentially expressed in the embryogenic callus (EC) of citrus and that the overexpression of *CsFUS3* in the recalcitrant callus can restore its embryogenic capacity. Additionally, *CsFUS3* is capable of resolving the embryonic defects associated with the *Arabidopsis fus3* mutant [135].

Researchers have cloned the *L1L* gene (*TcL1L*) from *Theobroma cacao*. In situ hybridization experiments revealed that *TcL1L* transcripts are mainly localized in different types of embryonic cells and expressed in immature gametes and somatic embryos. They have not, however, been detected in non-embryogenic calluses, indicating that their expression is specific to embryonic tissues [146]. Furthermore, the ectopic expression of *TcL1L* in *Arabidopsis* partially rescued the mutant phenotype associated with the *lec1* gene, indicating that *TcL1L* functions similarly to the *Arabidopsis LEC1* gene during zygotic embryogenesis. In subsequent experiments, Morgan et al. developed a system in *T. cacao* using a fusion of a transcription factor, *35S∷TcLEC2*-*GR*, and the glucocorticoid receptor (*GR*) activated by synthetic glucocorticoid dexamethasone (DEX) [147]. This fusion system successfully induced somatic embryogenesis from cocoa leaf tissues, representing a significant breakthrough. Andrews et al. utilized this system to greatly improve the efficiency of somatic embryogenesis and, ultimately, to generate transgenic cocoa plants with normal development, greatly enhancing the commercial value of cocoa trees [148].

When the *AtLEC2* gene was ectopically expressed in *Brassica napus*, somatic embryos were formed on the cotyledon petioles [130]. Guo et al. conducted experiments on tobacco plants with the ectopic expression of *AtLEC1* and *AtLEC2* genes, and the results showed that, under the stimulation of the glucocorticoid receptor-dexamethasone (GR-DEX) fusion system, *AtLEC2-GR* induced embryogenic callus and shoot formation in tobacco, whereas *LEC1* did not exhibit this ability [126]. Based on *AtLEC2-GR* transgenic tobacco, Ke Li et al. further improved the embryogenic callus induction rate and shoot regeneration capacity by overexpressing *AtIPT3*, *AtIPT7*, and *AtIPT9* under DEX culture conditions, demonstrating the important role of *LEC2* in somatic embryogenesis in tobacco [131].

Homologous genes of *AtLEC1* and *AtLEC2* have been identified in cassava and called *MeLEC1* and *MeLEC2*. An analysis of their expression during somatic embryogenesis in cassava revealed higher expression levels in somatic embryogenic (SE) tissues compared to differentiated mature tissues [142]. A series of experiments indicated that *MeLEC* genes play crucial roles in somatic embryogenesis in cassava. Recent research findings suggest that *MtLEC2* directly participates in somatic embryogenesis in *M. truncatula* [151].

The aforementioned studies indicate that *LEC* genes have emerged as key regulatory factors in controlling somatic embryogenesis and that they hold potential for application in plant regeneration.

## 7. Other Transcription Factor Genes Controlling Plant Embryogenesis

Some other transcription factor genes have also been found to promote embryogenesis, such as *SERK*, *WIND1*, and *LBD* [152,153] (Table 5). The somatic embryogenesis receptor-like kinase (*SERK*) protein, a receptor kinase involved in the regulation of somatic embryo development, was first identified in carrot cells [154]. Subsequently, it was found to play an important role in somatic embryogenesis in other species, such as *Arabidopsis*, *T. cacao*, *M. truncatula*, *T. aestivum*, and maize [155,156,157,158,159]. In a study with *Arabidopsis*, *AtSERK1* was found to be highly expressed in the early stages of embryogenesis. The overexpression of the *AtSERK1* gene in *Arabidopsis* resulted in a three–four-fold increase in somatic embryogenesis efficiency [155]. In *M. truncatul*, the *MtSERK1* gene was upregulated during embryogenesis, indicating its involvement in somatic embryogenesis in this species [157,160]. Similarly, *ZmSERK1* and *ZmSERK2* genes isolated from maize were found to be expressed during embryogenesis and were associated with hormone signaling and embryo development, suggesting their regulatory role in embryogenesis [159]. Therefore, the overexpression of the development-regulating factor *SERK* increases the occurrence of somatic embryogenesis and subsequently improves the efficiency of genetic transformation in plants.

The *AP2/ERF* transcription factor *WOUND INDUCE DEDIFFERENTIATION1* (*WIND1)* plays a crucial role in promoting the formation and regeneration of the plant callus [32,172]. The overexpression of the *AtWIND1* gene has been shown to enhance callus formation in *Arabidopsis*, tobacco, and tomatoes under hormone-free culture conditions [171]. A Dex-mediated *AtWIND1* induction system has also been studied. Researchers introduced the *35S:: AtWIND1-GR* vector into *Brassica napus* and tomatoes, which promoted callus formation and, subsequently, the development of shoots from the callus when cultured under ordinary, hormone-free conditions [170]. This further confirms the role of *WIND1* in cellular regeneration. Due to its ability to enhance plant regeneration, the interaction of *WIND1* with other developmental regulatory factors has been explored. The researchers hybridized *Arabidopsis* expressing *XVE-WIND1* (*WIND1* controlled by an inducible promoter) with *Arabidopsis* overexpressing *LEC2* using the *35S::LEC2-gr* structure. Apical meristems from the hypocotyls of yellowing seedlings were used as the explant to examine the induction effect. The results showed that when *LEC2* expression was induced using DEX alone, a limited callus formed around only the apical meristems and wound sites. When *WIND1* expression was induced using *ED* (17β-estradiol) alone, more callus formation occurred, but it did not lead to organ regeneration. However, when *WIND1* was induced followed by *LEC2* induction, the formation of an embryogenic callus was promoted in different tissues [170]. Subsequently, transferring the callus to hormone-free culture media promoted plant regeneration. In summary, the combination of *WIND1* and other developmental regulatory factors provides new insights into promoting explant regeneration.

The *LATERAL ORGAN BOUNDARIES DOMAIN* (*LBD)* gene family is a group of transcription factors specific to higher plants [22,173]. They contain a highly conserved *AS2/LOB* (*ASYMMETRIC LEAVES2/LATERAL ORGAN BOUNDARIES*) domain at the N-terminal, and they play important regulatory roles in various aspects of plant growth and development [174,175]. In the context of plant regeneration, *LBD* genes are crucial for embryogenesis and callus formation. In *Arabidopsis*, the ectopic expression of *LBD16*, *LBD17*, *LBD18*, and *LBD29* promotes callus formation, and these four *LBD* genes can also promote callus formation in *Arabidopsis*, even without exogenous plant hormones [22]. Subsequent studies have shown that an interaction occurs between the *Arabidopsis* basic region/leucine zipper motif 59 (*AtbZIP59*) transcription factor and *LBD*, forming a complex that directly regulates the transcription of the *FAD*-binding berberine (*FAD-BD*) gene and thereby promoting callus formation [24]. The *LBD* transcription factors are crucial regulatory factors in auxin-induced callus formation, and they provide insights into the mechanisms of plant embryogenesis.

The *AINTEGUMENTA*-*LIKE* (*AIL*) proteins represent a family of transcription factors characterized by the *APETALA2*/*ETHYLENE RESPONSE FACTOR* (*AP2*/*ERF*) domain. *Arabidopsis* harbors eight AIL transcription factor genes, including *AINTEGUMENTA* (*ANT*), *BABY BOOM* (*BBM*), and *PLETHORA* (*PLT*), all of which are extensively expressed in dividing meristematic tissues [43]. AIL/PLT genes play pivotal roles in establishing and maintaining meristematic tissues and initiating and sustaining organ growth.

It is a well-documented phenomenon that the ectopic expression of the BABY *BOOM*/*PLETHORA4* (*BBM*/*PLT4*) transcription factor, a member of the *AINTEGUMENTA*-*LIKE*/*PLETHORA* (*AIL*/*PLT*) clade, has the capacity to trigger somatic embryos [40,176,177]. Considering that somatic embryogenesis can facilitate regenerative processes in various breeding applications, this holds significant agricultural relevance. *PLETHORA* (*PLT*) transcription factors, members of the *APETALA2*/*ETHYLENE*-*RESPONSIVE ELEMENT BINDING PROTEIN* family, play crucial roles in post-embryonic development [178,179]. Merijn et al. conducted a series of experiments to prove that the ectopic overexpression of *PLT* promoted somatic embryogenesis [180]. In this investigation, it is substantiated that the collaborative action of *PLT2* and *BBM* redundantly facilitates the progression of the zygote into a developing embryo. The manifestation of these two genes becomes evident in the zygote shortly following fertilization. Additionally, along with *PLT7*, they exhibit expression in the apical cell after the inaugural asymmetric cell division. The absence of both *PLT2* and *BBM* culminates in embryonic arrest at the zygote/one-cell stage—a distinctive phenotype that remains unparalleled among various combinations of *PLT* single- and higher-order mutants.

The overexpression of *PLT* can induce cells to acquire pluripotency, which enables the gene to have multiple functions in plants; researchers can use this feature to improve plant regeneration efficiency, providing new ideas for plant regeneration and a theoretical basis for in vitro culture, the construction of transgenic plants, and molecular breeding.

## 8. Emerging Strategies for Plant Genetic Transformation and the Study of Exogenous Factors Enhancing Regeneration

Developmental regulatory factors, as well as certain exogenously applied metabolites, peptides, and/or small molecules, have been explored for their potential to promote cell fate transitions and enhance genetic transformation efficiency in plants.

Plant Sulfated Peptide (PSK) is a disulfated pentapeptide classified within the group of plant peptide growth factors [181]. Treatments with PSK have been demonstrated to stimulate the growth of callus tissues in rice and to induce somatic embryogenesis in species such as carrots, Japanese cedar (*Cryptomeria japonica*), and Japanese larch (*Larix leptolepis*) [182,183,184,185]. Recent research has revealed that, in the context of cedar somatic embryogenesis and plant regeneration systems, the addition of the plant small peptide hormone PSK to somatic embryo induction media significantly enhances the efficiency and duration of embryogenic induction for genotypes capable of somatic embryogenesis. Moreover, previously recalcitrant genotypes for somatic embryogenesis exhibited successful somatic embryo formation and plant regeneration under PSK treatment conditions. Phenotypic assessments, DCF and DAB staining, and H_2_O_2_ content measurements indicated that PSK treatment early in somatic embryo induction reduces peroxidase (PRX) transcriptional activity, maintains reactive oxygen species homeostasis, and enhances the expression of somatic embryogenesis-related genes like *WOX2*, ultimately promoting somatic embryogenesis in cedar [186].

The research conducted by Pil Joon Seo and his team unveiled that the exogenous application of adenosine monophosphate (AMP) enhances plant regenerative capacities [187]. A transcriptome analysis revealed a significant upregulation of genes involved in AMP synthesis in pluripotent callus tissues. A mutant analysis further demonstrated that the regenerative promotion effect of AMP is dependent on *PLETHORA* (*PLT*) genes, including *PLT3*, *PLT5*, and *PLT7*. Additionally, this study showcased the broad applicability of AMP in plant tissue culture and in improving regeneration capacities through protoplast regeneration in plants such as cabbage and tomatoes. In conclusion, our research indicates that AMP can be utilized to augment in vitro plant regeneration and that it is applicable across multiple plant species, representing a significant contribution to the field.

MicroRNA-encoded peptides (miPEPs) possess the capability to trigger their cognate microRNAs, thereby modulating plant growth and development. Furthermore, these peptides can be chemically synthesized and utilized as environmentally benign and harmless growth modulators, thus enhancing various agronomic attributes [188]. To explore the physiological function of miPEP in the development of poplar roots, researchers synthesized a root-specific miPEP and applied it to a poplar root regeneration medium. The results showed that miPEP166i was able to increase the number and length of adventitious roots compared with random sequence peptides [189].

**Figure 2:**
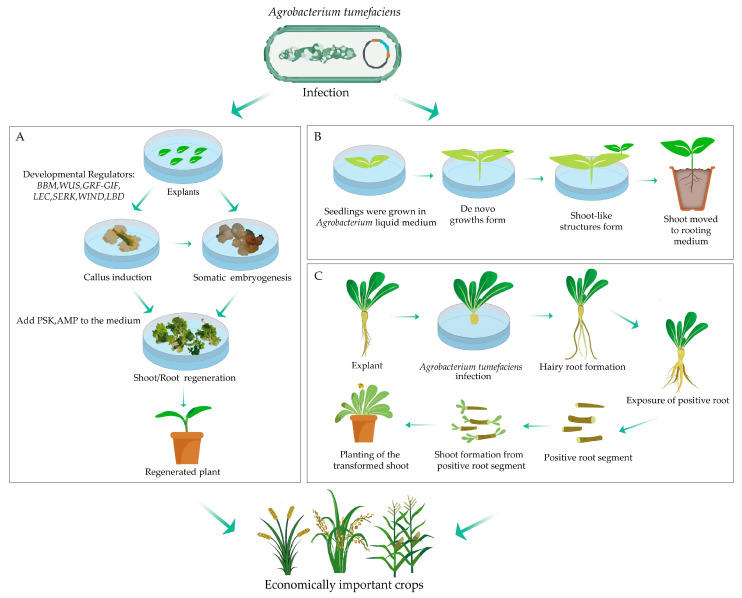
Strategies to improve regeneration and transformation efficiencies in *Agrobacterium*-mediated genetic transformation. (**A**) The application of developmental regulators such as *BBM*, *WUS*, *GRF-GIF*, *LEC*, *SERK*, *WIND*, and *LBD*, and small molecules such as PSK and AMP promotes plant regeneration and transformation efficiency in *Agrobacterium*-mediated genetic transformation. (**B**) Fast-TrACC method. The first step of the Fast-TrACC is to optimize *A. tumefaciens* cultures for gene transfer. Seedlings were germinated and co-cultured with the optimized A. tumefaciens strain. After about 2 weeks, dark-green growth began to form and eventually produced shoot-like structures. The shoot-like growth was then induced to form roots [190]. (**C**) CDB delivery system workflow. Seedlings 3–4 weeks old are cut as explants. Reporter genes or gene editing constructs are delivered to plant cells near the cut via *A. rhizogenes*. Hairy roots are formed after a few weeks. GFP-positive roots are cultured in sections to produce transgene-positive or gene-edited shoots [191].

In recent years, novel strategies have emerged for enhancing genetic transformation in plants. One such approach is the simultaneous expression of different developmental regulators (DRs) combined with gene-editing reagents. The Fast-Treated *Agrobacterium* Coculture (Fast-TrACC) method, for instance, facilitates the induction of transgenic and gene-edited meristematic tissues in plant seedlings. These tissues can generate flowers and seeds, thus enabling the transmission of transgenic and gene-edited traits to subsequent generations. Notably, gene editing in young shoots can occur directly in soil-grown plants, obviating the need for aseptic culture. Fast-TrACC holds promise for circumventing traditional tissue culture methods, thereby enhancing the efficiency of creating transgenic and gene-edited plants (Figure 2B) [190].

Another method, namely the Cut–Dip–Budding Delivery System (CBD), has been verified as a tissue culture-free approach for efficient genetic transformation or gene editing in species that were previously challenging to modify (Figure 2C). CBD utilizes a straightforward process of immersing explants in a specific suspension of *A. rhizogenes*, where the reporter gene or gene editing vector is delivered via *A. rhizogenes* to the plant cells near the cut site, which in turn results in the formation of a hairy root, and the positive root is sectioned and cultured to regenerate transgenic shoots, which are then cultivated to form a transgenic plant [191]. This method has made it possible to achieve genetic transformations that were once difficult or deemed impossible, although this method is currently only applicable to plants with a strong root-sucking ability and was successfully achieved by transforming root segments [192]. With ongoing exploration, it is anticipated that more species will undergo regeneration and genetic transformation through streamlined methods in the future, ultimately enhancing breeding efficiency.

## 9. Conclusions and Prospects

Somatic embryogenesis is a unique process in most plants’ life cycles that has become an instrumental tool in plant biotechnology. The aforementioned advancements indicate the feasibility of improving regeneration efficiency and genetic transformation efficacy by leveraging regeneration-associated genes, particularly in agricultural crops. These advancements have substantial potential for practical applications in crop production. However, the repertoire of currently employable regeneration-related genes is limited, and certain species with uncharacterized genotypes still present challenges for successful transformation. Moreover, although some studies have examined the regulatory pathways governing somatic embryogenesis, a complete elucidation of the underlying regulatory mechanisms remains elusive, and practical applications continue to be constrained.

A recent study conducting allelic gene-specific transcriptional analysis during shoot regeneration in hybrid poplar 84K (*Populus alba × P. tremula* var. *glandulosa* cv. 84K) unveiled the conservation and diversity of transcriptional regulation in the regeneration processes of poplar and *Arabidopsis*, providing valuable clues for the in-depth study of regeneration-related genes [193]. Additionally, the researchers mined alleles at the genome-wide level and used gene transcriptome and DNA methylation data in an integrated manner to analyze the molecular basis of the involvement of DNA methylation in the regulation of transcription processes during de novo shoot organogenesis [194]. Pointing out research directions for future research related to plant regeneration. These strides in technology have indicated that revealing the magnitude of cellular heterogeneity stands as an essential prerequisite for comprehending the fundamental principles and regulatory networks governing plant regeneration. The heightened resolution in transcriptional profiling achieved through high-throughput single-cell RNA sequencing (scRNA-seq) has notably enhanced the ability to compare regenerative-competent and non-competent cells in regenerating tissues [195]. These advancements serve as a foundational step in identifying progenitor cells and deducing transitions in cellular states.

Consequently, future efforts should entail broader research, focusing on developmental regulatory factors to enhance plant genetic transformation. For example, in-depth analyses of plant regeneration regulatory networks, coupled with cutting-edge bioinformatics techniques, can uncover additional embryogenesis-related transcription factors. Subsequent experimental verifications should encompass a wider range of plant species. Furthermore, capitalizing on the synergistic advantages offered by different combinations of developmental regulatory factors is essential. As more regeneration-related genes and mechanisms of action are identified and elucidated, the plant regeneration regulatory network will progressively become clearer, potentially resolving issues related to genotype limitations in plant genetic transformation. 

## Figures and Tables

**Table 1:** BBM genes from different plant species and their biological functions in plant regeneration.

Gene	Species Transformed	Biological Function	Ref.
*AtBBM*	*Arabidopsis thaliana*	Promote cell proliferation and somatic cell embryo formation	[40]
*AtBBM*	*Arabidopsis thaliana*	Promote somatic embryogenesis	[41]
*AtBBM*	*Arabidopsis thaliana*	Promote cell proliferation and somatic embryogenesis	[45]
*AtBBM*	*Arabidopsis thaliana* *Brassica napus* *Solanum lycopersicon*	Promote asexual embryo development	[46]
*AtBBM:GR*	*Arabidopsis thaliana*	Activation of complex developmental pathways for cell proliferation and cell growth	[44]
*BnBBM1/BnBBM2*	*Brassica napus*	Promote cell proliferation and morphogenesis during embryogenesis	[40]
*BnBBM*	*Capsicum annuum*	Promote somatic embryogenesis and improve regeneration efficiency	[47]
*CaBBM*	*Coffea arabica*	Involved in somatic embryogenesis	[48]
*GmBBM*	*Glycine max*	Promote somatic embryogenesis and embryo development	[49]
*LdBBM*	*Larix decidua*	Involved in somatic embryogenesis	[50]
*MaBBM1/MaBBM2*	*Musa acuminata*	Promote somatic embryogenesis	[51]
*PsASGR-BBML*	*Pennisetum glaucum*	Induction of apomixis	[52]
*RcBBM1 RcBBM2*	*Rosa canina*	Promote somatic embryogenesis and improve shoot regeneration efficiency	[53]
*TaBBM*	*Triticum aestivum*	Expressed during somatic embryogenesis	[54]
*TcBBM*	*Theobroma cacao*	Increase efficiency of somatic embryogenesis	[55]
*ZmBBM*	*Zea mays *(B73)*/Sorghum bicolor* (P898012)	Improve transformation efficiency	[56]
*ZmBBM*	*Zea mays* *Sorghum bicolor* *Saccharum officinarum* (v. CP01-1372) *Oryza sativa* ssp. *indica*	Increase transformation efficiency and promote plant regeneration	[57]
*ZmBBM*	*Zea mays*	Improve transformation efficiency	[58]

**Table 2:** WUS and WUS-related homeobox (WOX) transcription factors and their biological functions in plant regeneration.

Gene	Species Transformed	Biological Function	Ref.
*AtWUS*	*Arabidopsis thaliana*	Promote the vegetative-to-embryonic transition and somatic embryo formation	[67]
*AtWUS*	*Coffea canephora*	Increase in ectopic morphogenesis and somatic cell embryogenesis	[68]
*AtWUS*	*Capsicum chinense*	Promote the transition from an asexual to embryonic state	[69]
*AtWUS*	*Gossypium hirsutum*	Promote somatic embryogenesis and induce organogenesis	[70]
*AtWUS*	*Gossypium hirsutum*	Promote the formation of the embryogenic callus	[71]
*AtWUS*	*Medicago truncatula*	Increase callogenesis and embryogenesis	[72]
*AtWUS*	*Picea glauca*	Involved in somatic embryogenesis and somatic seedling growth	[73]
*AtWUS/WUS-P2A-BBM*	*Antirrhinum majus*	Induction of shoot regeneration and transformation efficiency	[12]
*AtWOX2/WOX8/WOX9*	*Arabidopsis thaliana*	Promote embryonic cell development	[65]
*AtWOX11/WOX12*	*Arabidopsis thaliana*	Induction of de novo root organogenesis	[34]
*AtWOX2/WOX9*	*Nicotiana tabacum*	Promote embryonic development	[74]
*AtWOX2/WOX8/WOX9*	*Nicotiana tabacum*	Promote regeneration from leaf segments and free cells	[75]
*CjWUS*	*Cryptomeria japonica*	Promote embryonic cell development	[76]
*LcWUS/LcWOX*	*Liriodendron hybrids*	Involved in somatic embryogenesis	[77]
*LdWOX2*	*Larix decidua*	Promote somatic embryogenesis	[50]
*MtWUS*	*Medicago truncatula*	Essential for callus and somatic embryo production	[78]
*MtWUS/MtWOX5*	*Medicago truncatula*	Expressed in calli with different embryogenic competence	[79]
*MtWOX11-like*	*Medicago truncatula*	Expressed during the early stages of somatic embryogenesis	[80]
*MtWOX9-1*	*Medicago truncatula*	Involved in somatic embryogenesis and stimulating somatic embryogenesis	[81]
*OcWUS/OcWOX4*	*Ocotea catharinensis*	Expression during somatic embryogenesis	[82]
*PaWOX8/9*	*Picea abies*	Expressed during the early stages of somatic embryogenesis	[83]
*PaWOX2*	*Picea abies*	Expressed during the early stages of somatic embryogenesis	[84]
*PpWOX2*	*Pinus pinaster*	Change the number and quality of embryos in cotyledon	[85]
*PpWOX2*	*Arabidopsis thaliana*	Promote somatic embryogenesis and organogenesis	[85]
*TaWOX5*	*Triticum aestivum*	Improve the transformation efficiency	[86]
*VvWOX2/VvWOX9*	*Vitis vinifera*	Expressed during the early stages of somatic embryogenesis	[87]
*VvWOX3/VvWOX11*	*Vitis vinifera*	Expresses at specific stages of somatic embryos (torpedo and cotyledonary)	[87]

**Table 3:** GRF/GRF-GIF and their biological functions in different plant species.

Gene	Species Transformed	Biological Function	Ref.
*AtGRF5*	*Citrullus lanatus*	Improve transformation efficiency	[111]
*AtGRF5*	*Beta vulgaris* ssp. *vulgaris*	Induction of new shoot formation and improve transformation efficiency	[112]
*AtGRF5/AtGRF6/AtGRF9*	*Brassica napus*	Promote callus proliferation	[112]
*AtGRF5/ZmGRF5-LIKE*	*Zea mays*	Promote somatic embryogenesis	[112]
*BnGRF5-LIKE*	*Brassica napus*	Increases in genetic transformation of the explant tissue	[112]
*ClGRF4-ClGIF1*	*Citrullus lanatus*	Improve transformation efficiency	[107]
*CsGRF3–CsGIF1*	*Cannabis sativa*	Improved regeneration efficiency	[106]
*GmGRF5-LIKE/AtGRF5*	*Glycine max*	Increase in genetic transformation of the explant tissue	[112]
*HaGRF5-LIKE/AtGRF5*	*Helianthus annuus*	Increase in genetic transformation of the explant tissue	[112]
*TaGRF4-GIF1*	*Triticum turgidum.* var. *durum* *Triticum aestivum* *Oryza sativa* *Triticale rimpau*	Improve the regeneration rate and genetic transformation efficiency	[105]
*TaGRF4-GIF1-ZmBBM*	*Zea mays*	Enhance regeneration efficiency	[113]
*VvGRF4-GIF1*	*Citrus reticulata Blanco*	Enhance regeneration efficiency	[105]
*GRF4-GIF*	*Lactuca* spp.	Enhance in vitro regeneration and agrobacterium-mediated transformation efficiencies	[114]

**Table 4:** *LECs* and their biological functions in plant regeneration.

Gene	Species	Biological Function	Ref.
*AtLEC1*	*Arabidopsis thaliana*	Involved in controlling late embryogenesis development	[122]
*AtLEC1*	*Arabidopsis thaliana*	Promote plant embryo development	[123]
*AtLEC1*	*Arabidopsis thaliana*	Promote epigenetic reprogramming during early embryogenesis	[124]
*AtLEC1* */FUS3*	*Arabidopsis thaliana*	Induction of dedifferentiation and development in somatic cell embryogenesis	[125]
*AtLEC1* */L1L*	*Arabidopsis thaliana*	Regulation of somatic embryogenesis	[116]
*AtLEC1/LEC2/* *AtFUS3*	*Arabidopsis thaliana*	Promote somatic embryogenesis	[41]
*AtLEC1/AtLEC2*	*Nicotiana tabacum*	Promote somatic embryogenesis	[126]
*AtLEC2*	*Arabidopsis thaliana*	Induction of cell dedifferentiation	[127]
*AtLEC2*	*Arabidopsis thaliana*	Promote embryogenic induction in somatic tissues	[128]
*AtLEC2*	*Arabidopsis thaliana*	Promote embryo development	[117]
*AtLEC2*	*Arabidopsis thaliana*	Induction of embryogenesis and somatic embryogenesis in asexual cells	[129]
*AtLEC2*	*Brassica napus*	Induction of somatic cell embryo formation in cotyledon petioles	[130]
*AtLEC2*	*Nicotiana tabacum*	Promote embryogenic callus formation and shoot regeneration	[131]
*AtLEC2/FUS3*	*Arabidopsis thaliana*	Involved in early embryogenesis and response to seasonal temperature changes	[132]
*AtLEC1/2;FUS3*	*Arabidopsis thaliana*	Promote somatic embryogenesis	[133]
*CaLEC*	*Capsicum annuum*	Involved in somatic embryogenesis	[134]
*CsFUS3*	*Citrus sinensis*	Promotes somatic embryogenesis	[135]
*CsL1L*	*Citrus sinensis*	Induction of embryo-like structures	[136]
*DcLEC1*	*Daucus carota*	Induction of zygotic and somatic embryogenesis	[137]
*GhLEC1*	*Gossypium hirsutum*	Regulation of somatic embryogenesis by regulating auxin homeostasis	[138]
*HaL1L*	*Helianthus annuus*	Produce ectopic embryo- and shoot-like structures	[139]
*HaL1L*	*Helianthus annuus × H. tuberosus*	Promote somatic embryogenesis	[140]
*MaLEC2*	*Musa paradisiaca*	Essential in the somatic embryogenesis	[141]
*MeLEC1/MeLEC2*	*Manihot esculenta*	Promote somatic embryogenesis	[142]
*MsLEC1*	*Medicago sativa*	Induction of somatic embryogenesis from leaf protoplast cells	[143]
*MtLEC1/L1L*	*Medicago truncatula*	Promote somatic embryogenesis	[144]
*SoLEC*	*Sugarcane* var. *Bululawang*	Regulation of somatic embryogenesis	[145]
*TcL1L*	*Theobroma cacao*	Expression in both zygotic and somatic embryogenesis processes	[146]
*TcLEC2*	*Theobroma cacao*	Promote somatic embryogenic induction in leaves	[147]
*TcLEC2*	*Theobroma cacao*	Promote somatic embryogenesis in leaf tissue	[148]
*ZmLEC1*	*Zea mays*	Promote zygotic and somatic embryogenesis	[149]

**Table 5:** Other transcription factors and their biological functions in plant regeneration.

Gene	Species Transformed	Biological Function	Ref.
*AtSERK1*	*Arabidopsis thaliana*	Increase in efficiency of somatic embryogenesis	[155]
*AaSERK*	*A.andraeanum*	Involved in somatic embryogenesis	[161]
*CitSERK1*	*Citrus unshiu*	Involved in somatic embryogenesis	[162]
*ClSERK*	*Cyrtochilum loxense*	Involved in somatic embryogenesis	[163]
*DlSERK1*	*Diospyros lotus*	Regulation of embryo development	[164]
*GmSERK1*	*Glycine max*	Promote somatic embryogenesis	[165]
*HaSERK*	*Helianthus annuus*	Promote somatic embryogenesis	[166]
*MtSERK1*	*Medicago truncatula*	Involved in somatic embryogenesis	[160]
*PhSERKL*	*Phoenix Dactylifera*	Regulation of somatic embryogenesis	[167]
*PpSERK*	*Poa pratensis*	Involved in embryogenesis	[168]
*ZmSERK*	*Zea mays*	Regulated the embryogenesis	[159]
*AtWIND1*	*Arabidopsis thaliana*	Promote callus formation	[169]
*AtWIND1*	*Brassica napus*	Enhance de novo shoot regeneration	[170]
*AtWIND1*	*Nicotiana tabacum*	Promote callus formation	[171]
*AtWIND1*	*Lycopersicon esculentum*	Promote callus formation	[171]
*AtLBD16/LBD29*	*Arabidopsis thaliana*	Regulation of callus formation	[34]
*AtLBD16/LBD17/LBD18/LBD29*	*Arabidopsis thaliana*	Trigger spontaneous callus formation	[22]
*AtbZIP59–LBD16*	*Arabidopsis thaliana*	Trigger autonomous callus formation	[24]

## Data Availability

The original contributions presented in the study are included in the article, further inquiries can be directed to the corresponding author.

## References

[1] Xu L., Huang H. (2014). Genetic and Epigenetic Controls of Plant Regeneration. Curr. Top. Dev. Biol..

[2] Altpeter F., Springer N.M., Bartley L.E., Blechl A.E., Brutnell T.P., Citovsky V., Conrad L.J., Gelvin S.B., Jackson D.P., Kausch A.P. (2016). Advancing Crop Transformation in the Era of Genome Editing. Plant Cell.

[3] Kausch A.P., Nelson-Vasilchik K., Hague J., Mookkan M., Quemada H., Dellaporta S., Fragoso C., Zhang Z.J. (2019). Edit at Will: Genotype Independent Plant Transformation in the Era of Advanced Genomics and Genome Editing. Plant Sci..

[4] Ramkumar T.R., Lenka S.K., Arya S.S., Bansal K.C. (2020). A Short History and Perspectives on Plant Genetic Transformation. Methods Mol. Biol..

[5] Han Z.-F., Hunter D.M., Sibbald S., Zhang J.-S., Tian L. (2013). Biological Activity of the *Tzs* Gene of Nopaline *Agrobacterium tumefaciens* GV3101 in Plant Regeneration and Genetic Transformation. MPMI.

[6] Nishimura A., Ashikari M., Lin S., Takashi T., Angeles E.R., Yamamoto T., Matsuoka M. (2005). Isolation of a Rice Regeneration Quantitative Trait Loci Gene and Its Application to Transformation Systems. Proc. Natl. Acad. Sci. USA.

[7] Shrawat A.K., Lörz H. (2006). Agrobacterium-Mediated Transformation of Cereals: A Promising Approach Crossing Barriers. Plant Biotechnol. J..

[8] Hiei Y., Ishida Y., Komari T. (2014). Progress of Cereal Transformation Technology Mediated by Agrobacterium Tumefaciens. Front. Plant Sci..

[9] Gordon-Kamm B., Sardesai N., Arling M., Lowe K., Hoerster G., Betts S., Jones A.T. (2019). Using Morphogenic Genes to Improve Recovery and Regeneration of Transgenic Plants. Plants.

[10] Méndez-Hernández H.A., Ledezma-Rodríguez M., Avilez-Montalvo R.N., Juárez-Gómez Y.L., Skeete A., Avilez-Montalvo J.R., De-la-Peña C., Loyola-Vargas V.M. (2019). Signaling Overview of Plant Somatic Embryogenesis. Front. Plant Sci..

[11] Maren N.A., Duan H., Da K., Yencho G.C., Ranney T.G., Liu W. (2022). Genotype-Independent Plant Transformation. Hortic. Res..

[12] Lian Z., Nguyen C.D., Liu L., Wang G., Chen J., Wang S., Yi G., Wilson S., Ozias-Akins P., Gong H. (2022). Application of Developmental Regulators to Improve in Planta or in Vitro Transformation in Plants. Plant Biotechnol. J..

[13] Braybrook S., Harada J. (2008). LECs Go Crazy in Embryo Development. Trends Plant Sci..

[14] Jha P., Kumar V. (2018). BABY BOOM (BBM): A Candidate Transcription Factor Gene in Plant Biotechnology. Biotechnol. Lett..

[15] Haberlandt G. Culturversuehe Mit Isolierten Pflanzenzellen. Plant Tissue Culture: 100 Years Since Gottlieb Haberlandt.

[16] Ikeuchi M., Ogawa Y., Iwase A., Sugimoto K. (2016). Plant Regeneration: Cellular Origins and Molecular Mechanisms. Development.

[17] Ikeuchi M., Sugimoto K., Iwase A. (2013). Plant Callus: Mechanisms of Induction and Repression[OPEN]. Plant Cell.

[18] Sugimoto K., Gordon S.P., Meyerowitz E.M. (2011). Regeneration in Plants and Animals: Dedifferentiation, Transdifferentiation, or Just Differentiation?. Trends Cell Biol..

[19] Ikeuchi M., Favero D.S., Sakamoto Y., Iwase A., Coleman D., Rymen B., Sugimoto K. (2019). Molecular Mechanisms of Plant Regeneration. Annu. Rev. Plant Biol..

[20] Sugimoto K., Jiao Y., Meyerowitz E.M. (2010). Arabidopsis Regeneration from Multiple Tissues Occurs via a Root Development Pathway. Dev. Cell.

[21] Fukaki H., Nakao Y., Okushima Y., Theologis A., Tasaka M. (2005). Tissue-Specific Expression of Stabilized SOLITARY-ROOT/IAA14 Alters Lateral Root Development in Arabidopsis. Plant J..

[22] Fan M., Xu C., Xu K., Hu Y. (2012). LATERAL ORGAN BOUNDARIES DOMAIN Transcription Factors Direct Callus Formation in Arabidopsis Regeneration. Cell Res..

[23] Pandey S.K., Lee H.W., Kim M.-J., Cho C., Oh E., Kim J. (2018). LBD18 Uses a Dual Mode of a Positive Feedback Loop to Regulate ARF Expression and Transcriptional Activity in Arabidopsis. Plant J..

[24] Xu C., Cao H., Zhang Q., Wang H., Xin W., Xu E., Zhang S., Yu R., Yu D., Hu Y. (2018). Control of Auxin-Induced Callus Formation by bZIP59–LBD Complex in Arabidopsis Regeneration. Nat. Plants.

[25] Lee H.W., Kim M.-J., Kim N.Y., Lee S.H., Kim J. (2013). LBD18 Acts as a Transcriptional Activator That Directly Binds to the EXPANSIN14 Promoter in Promoting Lateral Root Emergence of Arabidopsis. Plant J..

[26] Daimon Y., Takabe K., Tasaka M. (2003). The CUP-SHAPED COTYLEDON Genes Promote Adventitious Shoot Formation on Calli. Plant Cell Physiol..

[27] Kareem A., Durgaprasad K., Sugimoto K., Du Y., Pulianmackal A.J., Trivedi Z.B., Abhayadev P.V., Pinon V., Meyerowitz E.M., Scheres B. (2015). PLETHORA Genes Control Regeneration by a Two-Step Mechanism. Curr. Biol..

[28] Gallois J.-L., Nora F.R., Mizukami Y., Sablowski R. (2004). WUSCHEL Induces Shoot Stem Cell Activity and Developmental Plasticity in the Root Meristem. Genes Dev..

[29] Yang S., Poretska O., Sieberer T. (2018). ALTERED MERISTEM PROGRAM1 Restricts Shoot Meristem Proliferation and Regeneration by Limiting HD-ZIP III-Mediated Expression of RAP2.6L. Plant Physiol..

[30] Che P., Lall S., Nettleton D., Howell S.H. (2006). Gene Expression Programs during Shoot, Root, and Callus Development in Arabidopsis Tissue Culture. Plant Physiol..

[31] Shi B., Zhang C., Tian C., Wang J., Wang Q., Xu T., Xu Y., Ohno C., Sablowski R., Heisler M.G. (2016). Two-Step Regulation of a Meristematic Cell Population Acting in Shoot Branching in Arabidopsis. PLoS Genet..

[32] Iwase A., Harashima H., Ikeuchi M., Rymen B., Ohnuma M., Komaki S., Morohashi K., Kurata T., Nakata M., Ohme-Takagi M. (2017). WIND1 Promotes Shoot Regeneration through Transcriptional Activation of ENHANCER OF SHOOT REGENERATION1 in Arabidopsis. Plant Cell.

[33] Chen L., Tong J., Xiao L., Ruan Y., Liu J., Zeng M., Huang H., Wang J.-W., Xu L. (2016). YUCCA-Mediated Auxin Biogenesis Is Required for Cell Fate Transition Occurring during de Novo Root Organogenesis in Arabidopsis. J. Exp. Bot..

[34] Liu J., Sheng L., Xu Y., Li J., Yang Z., Huang H., Xu L. (2014). *WOX11* and *12* Are Involved in the First-Step Cell Fate Transition during de Novo Root Organogenesis in *Arabidopsis*. Plant Cell.

[35] Sheng L., Hu X., Du Y., Zhang G., Huang H., Scheres B., Xu L. (2017). Non-Canonical WOX11-Mediated Root Branching Contributes to Plasticity in Arabidopsis Root System Architecture. Development.

[36] Fehér A. (2015). Somatic Embryogenesis—Stress-Induced Remodeling of Plant Cell Fate. Biochim. Biophys. Acta.

[37] Gaj M.D., Trojanowska A., Ujczak A., Mędrek M., Kozioł A., Garbaciak B. (2006). Hormone-Response Mutants of *Arabidopsis thaliana* (L.) Heynh. Impaired in Somatic Embryogenesis. Plant Growth Regul..

[38] Braybrook S.A., Stone S.L., Park S., Bui A.Q., Le B.H., Fischer R.L., Goldberg R.B., Harada J.J. (2006). Genes Directly Regulated by LEAFY COTYLEDON2 Provide Insight into the Control of Embryo Maturation and Somatic Embryogenesis. Proc. Natl. Acad. Sci. USA.

[39] Zheng Y., Ren N., Wang H., Stromberg A.J., Perry S.E. (2009). Global Identification of Targets of the Arabidopsis MADS Domain Protein AGAMOUS-Like15. Plant Cell.

[40] Boutilier K., Offringa R., Sharma V.K., Kieft H., Ouellet T., Zhang L., Hattori J., Liu C.-M., van Lammeren A.A.M., Miki B.L.A. (2002). Ectopic Expression of BABY BOOM Triggers a Conversion from Vegetative to Embryonic Growth. Plant Cell.

[41] Horstman A., Li M., Heidmann I., Weemen M., Chen B., Muino J.M., Angenent G.C., Boutilier K. (2017). The BABY BOOM Transcription Factor Activates the LEC1-ABI3-FUS3-LEC2 Network to Induce Somatic Embryogenesis. Plant Physiol..

[42] Riechmann J.L., Meyerowitz E.M. (1998). The AP2/EREBP Family of Plant Transcription Factors. Biol. Chem..

[43] Nole-Wilson S., Tranby T.L., Krizek B.A. (2005). AINTEGUMENTA-like (AIL) Genes Are Expressed in Young Tissues and May Specify Meristematic or Division-Competent States. Plant Mol. Biol..

[44] Passarinho P., Ketelaar T., Xing M., van Arkel J., Maliepaard C., Hendriks M.W., Joosen R., Lammers M., Herdies L., den Boer B. (2008). BABY BOOM Target Genes Provide Diverse Entry Points into Cell Proliferation and Cell Growth Pathways. Plant Mol. Biol..

[45] Kulinska-Lukaszek K., Tobojka M., Adamiok A., Kurczynska E.U. (2012). Expression of the BBM Gene during Somatic Embryogenesis of *Arabidopsis thaliana*. Biol. Plant..

[46] Chen B., Maas L., Figueiredo D., Zhong Y., Reis R., Li M., Horstman A., Riksen T., Weemen M., Liu H. (2022). BABY BOOM Regulates Early Embryo and Endosperm Development. Proc. Natl. Acad. Sci. USA.

[47] Heidmann I., de Lange B., Lambalk J., Angenent G.C., Boutilier K. (2011). Efficient Sweet Pepper Transformation Mediated by the BABY BOOM Transcription Factor. Plant Cell Rep..

[48] Silva A.T., Barduche D., do Livramento K.G., Paiva L.V. (2015). A Putative BABY BOOM-like Gene (CaBBM) Is Expressed in Embryogenic Calli and Embryogenic Cell Suspension Culture of *Coffea arabica* L.. In Vitro Cell. Dev. Biol.-Plant.

[49] El Ouakfaoui S., Schnell J., Abdeen A., Colville A., Labbé H., Han S., Baum B., Laberge S., Miki B. (2010). Control of Somatic Embryogenesis and Embryo Development by AP2 Transcription Factors. Plant Mol. Biol..

[50] Rupps A., Raschke J., Rümmler M., Linke B., Zoglauer K. (2016). Identification of Putative Homologs of Larix Decidua to BABY BOOM (BBM), LEAFY COTYLEDON1 (LEC1), WUSCHEL-Related HOMEOBOX2 (WOX2) and SOMATIC EMBRYOGENESIS RECEPTOR-like KINASE (SERK) during Somatic Embryogenesis. Planta.

[51] Büyük İ., Aras S. (2017). Genome-Wide in Silico Identification, Characterization and Transcriptional Analysis of the Family of Growth-Regulating Factors in Common Bean (*Phaseolus vulgaris* L.) Subjected to Polyethylene Glycol-Induced Drought Stress. Arch. Biol. Sci..

[52] Conner J.A., Mookkan M., Huo H., Chae K., Ozias-Akins P. (2015). A Parthenogenesis Gene of Apomict Origin Elicits Embryo Formation from Unfertilized Eggs in a Sexual Plant. Proc. Natl. Acad. Sci. USA.

[53] Yang H.F., Kou Y.P., Gao B., Soliman T.M.A., Xu K.D., Ma N., Cao X., Zhao L.J. (2014). Identification and Functional Analysis of BABY BOOM Genes from *Rosa canina*. Biol. Plant.

[54] Bilichak A., Luu J., Jiang F., Eudes F. (2018). Identification of BABY BOOM Homolog in Bread Wheat. Agri Gene.

[55] Florez S.L., Erwin R.L., Maximova S.N., Guiltinan M.J., Curtis W.R. (2015). Enhanced Somatic Embryogenesis in Theobroma Cacao Using the Homologous BABY BOOM Transcription Factor. BMC Plant Biol..

[56] Mookkan M., Nelson-Vasilchik K., Hague J., Zhang Z.J., Kausch A.P. (2017). Selectable Marker Independent Transformation of Recalcitrant Maize Inbred B73 and Sorghum P898012 Mediated by Morphogenic Regulators BABY BOOM and WUSCHEL2. Plant Cell Rep..

[57] Lowe K., Wu E., Wang N., Hoerster G., Hastings C., Cho M.-J., Scelonge C., Lenderts B., Chamberlin M., Cushatt J. (2016). Morphogenic Regulators *Baby Boom* and *Wuschel* Improve Monocot Transformation. Plant Cell.

[58] Lowe K., La Rota M., Hoerster G., Hastings C., Wang N., Chamberlin M., Wu E., Jones T., Gordon-Kamm W. (2018). Rapid Genotype “Independent” *Zea mays* L. (Maize) Transformation via Direct Somatic Embryogenesis. In Vitro Cell. Dev. Biol.-Plant.

[59] Deng W., Luo K., Li Z., Yang Y. (2009). A Novel Method for Induction of Plant Regeneration via Somatic Embryogenesis. Plant Sci..

[60] Srinivasan C., Liu Z., Heidmann I., Supena E.D.J., Fukuoka H., Joosen R., Lambalk J., Angenent G., Scorza R., Custers J.B.M. (2006). Heterologous Expression of the BABY BOOM AP2/ERF Transcription Factor Enhances the Regeneration Capacity of Tobacco (*Nicotiana tabacum* L.). Planta.

[61] Bui L.T., Pandzic D., Youngstrom C.E., Wallace S., Irish E.E., Szövényi P., Cheng C.-L. (2017). A Fern *AINTEGUMENTA* Gene Mirrors *BABY BOOM* in Promoting Apogamy in *Ceratopteris richardii*. Plant J..

[62] Laux T., Mayer K.F., Berger J., Jürgens G. (1996). The WUSCHEL Gene Is Required for Shoot and Floral Meristem Integrity in Arabidopsis. Development.

[63] Mayer K.F., Schoof H., Haecker A., Lenhard M., Jürgens G., Laux T. (1998). Role of WUSCHEL in Regulating Stem Cell Fate in the Arabidopsis Shoot Meristem. Cell.

[64] Liu X., Kim Y.J., Müller R., Yumul R.E., Liu C., Pan Y., Cao X., Goodrich J., Chen X. (2011). AGAMOUS Terminates Floral Stem Cell Maintenance in Arabidopsis by Directly Repressing WUSCHEL through Recruitment of Polycomb Group Proteins. Plant Cell.

[65] Haecker A., Gross-Hardt R., Geiges B., Sarkar A., Breuninger H., Herrmann M., Laux T. (2004). Expression Dynamics of WOX Genes Mark Cell Fate Decisions during Early Embryonic Patterning in *Arabidopsis thaliana*. Development.

[66] West M.A.L., Harada J.J. (1993). Embryogenesis in Higher Plants: An Overview. Plant Cell.

[67] Zuo J., Niu Q.-W., Frugis G., Chua N.-H. (2002). The WUSCHEL Gene Promotes Vegetative-to-Embryonic Transition in Arabidopsis. Plant J..

[68] Arroyo-Herrera A., Ku Gonzalez A., Canche Moo R., Quiroz-Figueroa F.R., Loyola-Vargas V.M., Rodriguez-Zapata L.C., Burgeff D’Hondt C., Suárez-Solís V.M., Castaño E. (2008). Expression of WUSCHEL in *Coffea canephora* Causes Ectopic Morphogenesis and Increases Somatic Embryogenesis. Plant Cell Tissue Organ Cult..

[69] Solís-Ramos L.Y., González-Estrada T., Nahuath-Dzib S., Zapata-Rodriguez L.C., Castaño E. (2009). Overexpression of WUSCHEL in *C. chinense* Causes Ectopic Morphogenesis. Plant Cell Tissue Organ Cult..

[70] Bouchabké-Coussa O., Obellianne M., Linderme D., Montes E., Maia-Grondard A., Vilaine F., Pannetier C. (2013). Wuschel Overexpression Promotes Somatic Embryogenesis and Induces Organogenesis in Cotton (*Gossypium hirsutum* L.) Tissues Cultured in Vitro. Plant Cell Rep..

[71] Zheng W., Zhang X., Yang Z., Wu J., Li F., Duan L., Liu C., Lu L., Zhang C., Li F. (2014). AtWuschel Promotes Formation of the Embryogenic Callus in *Gossypium hirsutum*. PLoS ONE.

[72] Kadri A., Grenier De March G., Guerineau F., Cosson V., Ratet P. (2021). WUSCHEL Overexpression Promotes Callogenesis and Somatic Embryogenesis in Medicago *Truncatula gaertn*. Plants.

[73] Klimaszewska K., Pelletier G., Overton C., Stewart D., Rutledge R.G. (2010). Hormonally Regulated Overexpression of Arabidopsis WUS and Conifer LEC1 (CHAP3A) in Transgenic White Spruce: Implications for Somatic Embryo Development and Somatic Seedling Growth. Plant Cell Rep..

[74] Zhou X., Guo Y., Zhao P., Sun M. (2018). Comparative Analysis of WUSCHEL-Related Homeobox Genes Revealed Their Parent-of-Origin and Cell Type-Specific Expression Pattern During Early Embryogenesis in Tobacco. Front. Plant Sci..

[75] Kyo M., Maida K., Nishioka Y., Matsui K. (2018). Coexpression of *WUSCHEL Related Homeobox* (*WOX*) *2* with *WOX8* or *WOX9* Promotes Regeneration from Leaf Segments and Free Cells in *Nicotiana tabacum* L.. Plant Biotechnol..

[76] Izuno A., Maruyama T.E., Ueno S., Ujino-Ihara T., Moriguchi Y. (2020). Genotype and Transcriptome Effects on Somatic Embryogenesis in Cryptomeria Japonica. PLoS ONE.

[77] Long X., Zhang J., Wang D., Weng Y., Liu S., Li M., Hao Z., Cheng T., Shi J., Chen J. (2023). Expression Dynamics of WOX Homeodomain Transcription Factors during Somatic Embryogenesis in *Liriodendron* Hybrids. For. Res..

[78] Chen S.-K., Kurdyukov S., Kereszt A., Wang X.-D., Gresshoff P.M., Rose R.J. (2009). The Association of Homeobox Gene Expression with Stem Cell Formation and Morphogenesis in Cultured *Medicago truncatula*. Planta.

[79] Orłowska A., Kępczyńska E. (2018). Identification of Polycomb Repressive Complex1, Trithorax Group Genes and Their Simultaneous Expression with WUSCHEL, WUSCHEL-Related Homeobox5 and SHOOT MERISTEMLESS during the Induction Phase of Somatic Embryogenesis in *Medicago truncatula* Gaertn. Plant Cell Tissue Organ Cult..

[80] Tvorogova V.E., Lebedeva M.A., Lutova L.A. (2015). Expression of the WOX and PIN Genes in the Course of Somatic and Zygotic Embryogenesis of a *Medicago truncatula*. Genetika.

[81] Tvorogova V.E., Fedorova Y.A., Potsenkovskaya E.A., Kudriashov A.A., Efremova E.P., Kvitkovskaya V.A., Wolabu T.W., Zhang F., Tadege M., Lutova L.A. (2019). The WUSCHEL-Related Homeobox Transcription Factor MtWOX9-1 Stimulates Somatic Embryogenesis in *Medicago truncatula*. Plant Cell Tissue Organ Cult..

[82] Santa-Catarina C., de Oliveira R.R., Cutri L., Floh E.I.S., Dornelas M.C. (2012). WUSCHEL-Related Genes Are Expressed during Somatic Embryogenesis of the Basal Angiosperm *Ocotea catharinensis* Mez. (Lauraceae). Trees.

[83] Zhu T., Moschou P.N., Alvarez J.M., Sohlberg J.J., von Arnold S. (2014). WUSCHEL-RELATED HOMEOBOX 8/9 Is Important for Proper Embryo Patterning in the Gymnosperm Norway Spruce. J. Exp. Bot..

[84] Zhu T., Moschou P.N., Alvarez J.M., Sohlberg J.J., von Arnold S. (2016). WUSCHEL-RELATED HOMEOBOX 2 Is Important for Protoderm and Suspensor Development in the Gymnosperm Norway Spruce. BMC Plant Biol..

[85] Hassani S.B., Trontin J.-F., Raschke J., Zoglauer K., Rupps A. (2022). Constitutive Overexpression of a Conifer WOX2 Homolog Affects Somatic Embryo Development in Pinus Pinaster and Promotes Somatic Embryogenesis and Organogenesis in Arabidopsis Seedlings. Front. Plant Sci..

[86] Wang K., Shi L., Liang X., Zhao P., Wang W., Liu J., Chang Y., Hiei Y., Yanagihara C., Du L. (2022). The Gene TaWOX5 Overcomes Genotype Dependency in Wheat Genetic Transformation. Nat. Plants.

[87] Gambino G., Minuto M., Boccacci P., Perrone I., Vallania R., Gribaudo I. (2011). Characterization of Expression Dynamics of WOX Homeodomain Transcription Factors during Somatic Embryogenesis in Vitis Vinifera. J. Exp. Bot..

[88] Ramírez-Mosqueda M.A. (2022). Overview of Somatic Embryogenesis. Methods Mol. Biol..

[89] Chugh A., Khurana P. (2002). Gene Expression during Somatic Embryogenesis—Recent Advances. Current Sci..

[90] Costanzo E., Trehin C., Vandenbussche M. (2014). The Role of WOX Genes in Flower Development. Ann. Bot.

[91] Breuninger H., Rikirsch E., Hermann M., Ueda M., Laux T. (2008). Differential Expression of WOX Genes Mediates Apical-Basal Axis Formation in the Arabidopsis Embryo. Dev. Cell..

[92] Wu X., Chory J., Weigel D. (2007). Combinations of WOX Activities Regulate Tissue Proliferation during Arabidopsis Embryonic Development. Dev. Biol..

[93] Wan Q., Zhai N., Xie D., Liu W., Xu L. (2023). WOX11: The Founder of Plant Organ Regeneration. Cell Regen..

[94] Pan J., Zhao F., Zhang G., Pan Y., Sun L., Bao N., Qin P., Chen L., Yu J., Zhang Y. (2019). Control of de Novo Root Regeneration Efficiency by Developmental Status of Arabidopsis Leaf Explants. J. Genet. Genom..

[95] Hu B., Zhang G., Liu W., Shi J., Wang H., Qi M., Li J., Qin P., Ruan Y., Huang H. (2017). Divergent Regeneration-Competent Cells Adopt a Common Mechanism for Callus Initiation in Angiosperms. Regeneration.

[96] Sarkar A.K., Luijten M., Miyashima S., Lenhard M., Hashimoto T., Nakajima K., Scheres B., Heidstra R., Laux T. (2007). Conserved Factors Regulate Signalling in *Arabidopsis thaliana* Shoot and Root Stem Cell Organizers. Nature.

[97] Zhao S., Jiang Q.-T., Ma J., Zhang X.-W., Zhao Q.-Z., Wang X.-Y., Wang C.-S., Cao X., Lu Z.-X., Zheng Y.-L. (2014). Characterization and Expression Analysis of WOX5 Genes from Wheat and Its Relatives. Gene.

[98] Wang N., Arling M., Hoerster G., Ryan L., Wu E., Lowe K., Gordon-Kamm W., Jones T.J., Chilcoat N.D., Anand A. (2020). An Efficient Gene Excision System in Maize. Front. Plant Sci..

[99] van der Knaap E., Kim J.H., Kende H. (2000). A Novel Gibberellin-Induced Gene from Rice and Its Potential Regulatory Role in Stem Growth. Plant Physiol..

[100] Kim J.H., Choi D., Kende H. (2003). The AtGRF Family of Putative Transcription Factors Is Involved in Leaf and Cotyledon Growth in *Arabidopsis*. Plant J..

[101] Choi D., Kim J.H., Kende H. (2004). Whole Genome Analysis of the OsGRF Gene Family Encoding Plant-Specific Putative Transcription Activators in Rice (*Oryza sativa* L.). Plant Cell Physiol..

[102] Lee B.H., Ko J.-H., Lee S., Lee Y., Pak J.-H., Kim J.H. (2009). The Arabidopsis GRF-INTERACTING FACTOR Gene Family Performs an Overlapping Function in Determining Organ Size as Well as Multiple Developmental Properties. Plant Physiol..

[103] Omidbakhshfard M.A., Proost S., Fujikura U., Mueller-Roeber B. (2015). Growth-Regulating Factors (GRFs): A Small Transcription Factor Family with Important Functions in Plant Biology. Mol. Plant.

[104] Lee B.H., Wynn A.N., Franks R.G., Hwang Y., Lim J., Kim J.H. (2014). The *Arabidopsis thaliana* GRF-INTERACTING FACTOR Gene Family Plays an Essential Role in Control of Male and Female Reproductive Development. Dev. Biol..

[105] Debernardi J.M., Tricoli D.M., Ercoli M.F., Hayta S., Ronald P., Palatnik J.F., Dubcovsky J. (2020). A GRF–GIF Chimeric Protein Improves the Regeneration Efficiency of Transgenic Plants. Nat. Biotechnol..

[106] Zhang X., Xu G., Cheng C., Lei L., Sun J., Xu Y., Deng C., Dai Z., Yang Z., Chen X. (2021). Establishment of an Agrobacterium-Mediated Genetic Transformation and CRISPR/Cas9-Mediated Targeted Mutagenesis in Hemp (*Cannabis sativa* L.). Plant Biotechnol. J..

[107] Feng Q., Xiao L., He Y., Liu M., Wang J., Tian S., Zhang X., Yuan L. (2021). Highly Efficient, Genotype-independent Transformation and Gene Editing in Watermelon (*Citrullus lanatus*) Using a Chimeric *ClGRF4-GIF1* Gene. JIPB.

[108] Kim J.H. (2019). Biological Roles and an Evolutionary Sketch of the GRF-GIF Transcriptional Complex in Plants. BMB Rep..

[109] Hoe Kim J., Tsukaya H. (2015). Regulation of Plant Growth and Development by the GROWTH-REGULATING FACTOR and GRF-INTERACTING FACTOR Duo. EXBOTJ.

[110] Liebsch D., Palatnik J.F. (2020). MicroRNA miR396, GRF Transcription Factors and GIF Co-Regulators: A Conserved Plant Growth Regulatory Module with Potential for Breeding and Biotechnology. Curr. Opin. Plant Biol..

[111] Pan W., Cheng Z., Han Z., Yang H., Zhang W., Zhang H. (2022). Efficient Genetic Transformation and CRISPR/Cas9-Mediated Genome Editing of Watermelon Assisted by Genes Encoding Developmental Regulators. J. Zhejiang Univ. Sci. B.

[112] Kong J., Martin-Ortigosa S., Finer J., Orchard N., Gunadi A., Batts L.A., Thakare D., Rush B., Schmitz O., Stuiver M. (2020). Overexpression of the Transcription Factor GROWTH-REGULATING FACTOR5 Improves Transformation of Dicot and Monocot Species. Front. Plant Sci..

[113] Chen Z., Debernardi J.M., Dubcovsky J., Gallavotti A. (2022). The Combination of Morphogenic Regulators BABY BOOM and GRF-GIF Improves Maize Transformation Efficiency. Plant Biol..

[114] Bull T., Debernardi J., Reeves M., Hill T., Bertier L., Van Deynze A., Michelmore R. (2023). GRF–GIF Chimeric Proteins Enhance in Vitro Regeneration and Agrobacterium-Mediated Transformation Efficiencies of Lettuce (*Lactuca* Spp.). Plant Cell Rep.

[115] Yu Y., Yu H., Peng J., Yao W.J., Wang Y.P., Zhang F.L., Wang S.R., Zhao Y., Zhao X.Y., Zhang X.S. (2023). Enhancing Wheat Regeneration and Genetic Transformation through Overexpression of TaLAX1. Plant Commun..

[116] Kwong R.W., Bui A.Q., Lee H., Kwong L.W., Fischer R.L., Goldberg R.B., Harada J.J. (2003). LEAFY COTYLEDON1-LIKE Defines a Class of Regulators Essential for Embryo Development. Plant Cell.

[117] Stone S.L., Kwong L.W., Yee K.M., Pelletier J., Lepiniec L., Fischer R.L., Goldberg R.B., Harada J.J. (2001). *LEAFY COTYLEDON2* Encodes a B3 Domain Transcription Factor That Induces Embryo Development. Proc. Natl. Acad. Sci. USA.

[118] Harada J.J. (2001). Role of Arabidopsis LEAFY COTYLEDON Genes in Seed Development. J. Plant Physiol..

[119] Yeung E.C., Meinke D.W. (1993). Embryogenesis in Angiosperms: Development of the Suspensor. Plant Cell.

[120] Meinke D.W., Franzmann L.H., Nickle T.C., Yeung E.C. (1994). Leafy Cotyledon Mutants of Arabidopsis. Plant Cell.

[121] Keith K., Kraml M., Dengler N.G., McCourt P. (1994). Fusca3: A Heterochronic Mutation Affecting Late Embryo Development in Arabidopsis. Plant Cell.

[122] West M.A.L., Yee K.M., Danao J., Zimmerman J.L., Fischer R.L., Goldberg R.B., Harada J.J. (1994). LEAFY COTYLEDON1 Is an Essential Regulator of Late Embryogenesis and Cotyledon Identity in Arabidopsis. Plant Cell.

[123] Suzuki M., Wang H.H.-Y., McCarty D.R. (2007). Repression of the *LEAFY COTYLEDON 1/B3* Regulatory Network in Plant Embryo Development by *VP1*/*ABSCISIC ACID INSENSITIVE 3*—*LIKE* B3 Genes. Plant Physiol..

[124] Tao Z., Shen L., Gu X., Wang Y., Yu H., He Y. (2017). Embryonic Epigenetic Reprogramming by a Pioneer Transcription Factor in Plants. Nature.

[125] Ledwoń A., Gaj M.D. (2011). LEAFY COTYLEDON1, FUSCA3 Expression and Auxin Treatment in Relation to Somatic Embryogenesis Induction in Arabidopsis. Plant Growth Regul..

[126] Guo F., Liu C., Xia H., Bi Y., Zhao C., Zhao S., Hou L., Li F., Wang X. (2013). Induced Expression of AtLEC1 and AtLEC2 Differentially Promotes Somatic Embryogenesis in Transgenic Tobacco Plants. PLoS ONE.

[127] Stone S.L., Braybrook S.A., Paula S.L., Kwong L.W., Meuser J., Pelletier J., Hsieh T.-F., Fischer R.L., Goldberg R.B., Harada J.J. (2008). *Arabidopsis* LEAFY COTYLEDON2 Induces Maturation Traits and Auxin Activity: Implications for Somatic Embryogenesis. Proc. Natl. Acad. Sci. USA.

[128] Wójcikowska B., Jaskóła K., Gąsiorek P., Meus M., Nowak K., Gaj M.D. (2013). LEAFY COTYLEDON2 (LEC2) Promotes Embryogenic Induction in Somatic Tissues of Arabidopsis, via YUCCA-Mediated Auxin Biosynthesis. Planta.

[129] Lotan T., Ohto M., Yee K.M., West M.A.L., Lo R., Kwong R.W., Yamagishi K., Fischer R.L., Goldberg R.B., Harada J.J. (1998). Arabidopsis LEAFY COTYLEDON1 Is Sufficient to Induce Embryo Development in Vegetative Cells. Cell.

[130] Belide S., Zhou X.-R., Kennedy Y., Lester G., Shrestha P., Petrie J.R., Singh S.P. (2013). Rapid Expression and Validation of Seed-Specific Constructs in Transgenic LEC2 Induced Somatic Embryos of Brassica Napus. Plant Cell Tissue Organ Cult..

[131] Li K., Wang J., Liu C., Li C., Qiu J., Zhao C., Xia H., Ma C., Wang X., Li P. (2019). Expression of AtLEC2 and AtIPTs Promotes Embryogenic Callus Formation and Shoot Regeneration in Tobacco. BMC Plant Biol.

[132] Tao Z., Hu H., Luo X., Jia B., Du J., He Y. (2019). Embryonic Resetting of the Parental Vernalized State by Two B3 Domain Transcription Factors in Arabidopsis. Nat. Plants.

[133] Gaj M.D., Zhang S., Harada J.J., Lemaux P.G. (2005). Leafy Cotyledon Genes Are Essential for Induction of Somatic Embryogenesis of Arabidopsis. Planta.

[134] Irikova T., Grozeva S., Denev I. (2012). Identification of BABY BOOM and LEAFY COTYLEDON Genes in Sweet Pepper (*Capsicum annuum* L.) Genome by Their Partial Gene Sequences. Plant Growth Regul..

[135] Liu Z., Ge X.-X., Qiu W.-M., Long J.-M., Jia H.-H., Yang W., Dutt M., Wu X.-M., Guo W.-W. (2018). Overexpression of the CsFUS3 Gene Encoding a B3 Transcription Factor Promotes Somatic Embryogenesis in Citrus. Plant Sci..

[136] Zhu S., Wang J., Ye J., Zhu A.-D., Guo W., Deng X. (2014). Isolation and Characterization of LEAFY COTYLEDON 1-LIKE Gene Related to Embryogenic Competence in Citrus Sinensis. Plant Cell Tissue Organ Cult..

[137] Yazawa K., Takahata K., Kamada H. (2004). Isolation of the Gene Encoding Carrot Leafy Cotyledon1 and Expression Analysis during Somatic and Zygotic Embryogenesis. Plant Physiol. Biochem..

[138] Min L., Hu Q., Li Y., Xu J., Ma Y., Zhu L., Yang X., Zhang X. (2015). LEAFY COTYLEDON1-CASEIN KINASE I-TCP15-PHYTOCHROME INTERACTING FACTOR4 Network Regulates Somatic Embryogenesis by Regulating Auxin Homeostasis. Plant Physiol..

[139] Fambrini M., Durante C., Cionini G., Geri C., Giorgetti L., Michelotti V., Salvini M., Pugliesi C. (2006). Characterization of LEAFY COTYLEDON1-LIKE Gene in *Helianthus annuus* and Its Relationship with Zygotic and Somatic Embryogenesis. Dev. Genes Evol..

[140] Chiappetta A., Fambrini M., Petrarulo M., Rapparini F., Michelotti V., Bruno L., Greco M., Baraldi R., Salvini M., Pugliesi C. (2009). Ectopic Expression of LEAFY COTYLEDON1-LIKE Gene and Localized Auxin Accumulation Mark Embryogenic Competence in Epiphyllous Plants of *Helianthus annuus* × *H. Tuberosus*. Ann. Bot..

[141] Shivani, Awasthi P., Sharma V., Kaur N., Kaur N., Pandey P., Tiwari S. (2017). Genome-Wide Analysis of Transcription Factors during Somatic Embryogenesis in Banana (*Musa* Spp.) Cv. Grand Naine. PLoS ONE.

[142] Brand A., Quimbaya M., Tohme J., Chavarriaga-Aguirre P. (2019). Arabidopsis LEC1 and LEC2 Orthologous Genes Are Key Regulators of Somatic Embryogenesis in Cassava. Front. Plant Sci..

[143] Domoki M., Györgyey J., Bíró J., Pasternak T.P., Zvara Á., Bottka S., Puskás L.G., Dudits D., Fehér A. (2006). Identification and Characterization of Genes Associated with the Induction of Embryogenic Competence in Leaf-Protoplast-Derived Alfalfa Cells. Acta (BBA)—Gene Struct. Expr..

[144] Orłowska A., Igielski R., Łagowska K., Kępczyńska E. (2017). Identification of LEC1, L1L and Polycomb Repressive Complex 2 Genes and Their Expression during the Induction Phase of *Medicago truncatula* Gaertn. Somatic Embryogenesis. Plant Cell Tissue Organ Cult..

[145] Maulidiya A.U.K., Sugiharto B., Dewanti P., Handoyo T. (2020). Expression of Somatic Embryogenesis-Related Genes in Sugarcane (*Saccharum officinarum* L.). J. Crop Sci. Biotechnol..

[146] Alemanno L., Devic M., Niemenak N., Sanier C., Guilleminot J., Rio M., Verdeil J.-L., Montoro P. (2008). Characterization of Leafy Cotyledon1-like during Embryogenesis in *Theobroma cacao* L.. Planta.

[147] Shires M.E., Florez S.L., Lai T.S., Curtis W.R. (2017). Inducible Somatic Embryogenesis in Theobroma Cacao Achieved Using the DEX-Activatable Transcription Factor-Glucocorticoid Receptor Fusion. Biotechnol. Lett..

[148] Fister A.S., Landherr L., Perryman M., Zhang Y., Guiltinan M.J., Maximova S.N. (2018). Glucocorticoid Receptor-Regulated TcLEC2 Expression Triggers Somatic Embryogenesis in Theobroma Cacao Leaf Tissue. PLoS ONE.

[149] Zhang S., Wong L., Meng L., Lemaux P. (2002). Similarity of Expression Patterns of Knotted1 and ZmLEC1 during Somatic and Zygotic Embryogenesis in Maize (*Zea mays* L.). Planta.

[150] Kumar V., Jha P., Van Staden J. (2020). LEAFY COTYLEDONs (LECs): Master Regulators in Plant Embryo Development. Plant Cell Tissue Organ Cult..

[151] Barreto H.G., Ságio S.A., Chalfun-Júnior A., Fevereiro P., Benedito V.A. (2019). Transcriptional Profiling of the AFL Subfamily of B3-Type Transcription Factors during the in Vitro Induction of Somatic Embryogenesis in the Model Legume *Medicago truncatula*. Plant Cell Tissue Organ Cult..

[152] Pandey D.K., Chaudhary B. (2014). Role of Plant Somatic Embryogenesis Receptor Kinases (SERKs) in Cell-to-Embryo Transitional Activity: Key at Novel Assorted Structural Subunits. AJPS.

[153] Zhou C., Guo J., Feng Z., Cui X., Zhu J. (2012). Molecular Characterization of a Novel AP2 Transcription Factor ThWIND1-L from *Thellungiella halophila*. Plant Cell Tissue Organ Cult..

[154] Schmidt E.D., Guzzo F., Toonen M.A., de Vries S.C. (1997). A Leucine-Rich Repeat Containing Receptor-like Kinase Marks Somatic Plant Cells Competent to Form Embryos. Development.

[155] Hecht V., Vielle-Calzada J.-P., Hartog M.V., Schmidt E.D.L., Boutilier K., Grossniklaus U., de Vries S.C. (2001). The Arabidopsis *Somatic Embryogenesis Receptor Kinase 1* Gene Is Expressed in Developing Ovules and Embryos and Enhances Embryogenic Competence in Culture. Plant Physiol..

[156] de Oliveira Santos M., Romano E., Yotoko K.S.C., Tinoco M.L.P., Dias B.B.A., Aragão F.J.L. (2005). Characterisation of the Cacao Somatic Embryogenesis Receptor-like Kinase (SERK) Gene Expressed during Somatic Embryogenesis. Plant Sci..

[157] Nolan K.E., Irwanto R.R., Rose R.J. (2003). Auxin Up-Regulates MtSERK1 Expression in Both *Medicago truncatula* Root-Forming and Embryogenic Cultures. Plant Physiol..

[158] Singla B., Khurana J.P., Khurana P. (2008). Characterization of Three Somatic Embryogenesis Receptor Kinase Genes from Wheat, *Triticum aestivum*. Plant Cell Rep..

[159] Zhang S., Liu X., Lin Y., Xie G., Fu F., Liu H., Wang J., Gao S., Lan H., Rong T. (2011). Characterization of a ZmSERK Gene and Its Relationship to Somatic Embryogenesis in a Maize Culture. Plant Cell Tissue Organ Cult..

[160] Nolan K.E., Kurdyukov S., Rose R.J. (2011). Characterisation of the Legume SERK-NIKgene Superfamily Including Splice Variants: Implications for Development and Defence. BMC Plant Biol..

[161] Tian N. (2015). Clone and Expression of AaSERK Gene in Anthurium Andraeanum’s Somatic Embryogenesis. Acta Bot. Boreali-Occident. Sin..

[162] Shimada T., Hirabayashi T., Endo T., Fujii H., Kita M., Omura M. (2005). Isolation and Characterization of the Somatic Embryogenesis Receptor-like Kinase Gene Homologue (CitSERK1) from Citrus Unshiu Marc. Sci. Hortic..

[163] Santa-Catarina C., Hanai L.R., Dornelas M.C., Viana A.M., Floh E.I.S. (2004). SERK Gene Homolog Expression, Polyamines and Amino Acids Associated with Somatic Embryogenic Competence of *Ocotea catharinensis* Mez. (Lauraceae). Plant Cell Tissue Organ Cult..

[164] Zhou R., Wang Y., Zhang X., Jia F., Liu Y. (2022). Cloning and Expression Analysis of *SERK1* Gene in *Diospyros lotus*. Open Life Sci..

[165] Yang C., Zhao T., Yu D., Gai J. (2011). Isolation and Functional Characterization of a SERK Gene from Soybean (*Glycine max* (L.) Merr.). Plant Mol. Biol. Rep..

[166] Thomas C., Meyer D., Himber C., Steinmetz A. (2004). Spatial Expression of a Sunflower SERK Gene during Induction of Somatic Embryogenesis and Shoot Organogenesis. Plant Physiol. Biochem..

[167] Rekik I., Drira N., Grubb D., Elleuch A. (2015). Molecular Characterization and Evolution Studies of a SERK like Gene Transcriptionally Induced during Somatic Embryogenesis in Phoenix Dactylifera L v Deglet Nour. Genetika.

[168] Albertini E., Marconi G., Reale L., Barcaccia G., Porceddu A., Ferranti F., Falcinelli M. (2005). SERK and APOSTART. Candidate Genes for Apomixis in Poa Pratensis1[w]. Plant Physiol..

[169] Iwase A., Kondo Y., Laohavisit A., Takebayashi A., Ikeuchi M., Matsuoka K., Asahina M., Mitsuda N., Shirasu K., Fukuda H. (2021). WIND Transcription Factors Orchestrate Wound-induced Callus Formation, Vascular Reconnection and Defense Response in Arabidopsis. New Phytol..

[170] Iwase A., Mita K., Nonaka S., Ikeuchi M., Koizuka C., Ohnuma M., Ezura H., Imamura J., Sugimoto K. (2015). WIND1-Based Acquisition of Regeneration Competency in Arabidopsis and Rapeseed. J. Plant Res..

[171] Iwase A., Mitsuda N., Ikeuchi M., Ohnuma M., Koizuka C., Kawamoto K., Imamura J., Ezura H., Sugimoto K. (2013). *Arabidopsis* WIND1 Induces Callus Formation in Rapeseed, Tomato, and Tobacco. Plant Signal. Behav..

[172] Iwase A., Ohme-Takagi M., Sugimoto K. (2011). WIND1: A Key Molecular Switch for Plant Cell Dedifferentiation. Plant Signal. Behav..

[173] Husbands A., Bell E.M., Shuai B., Smith H.M.S., Springer P.S. (2007). LATERAL ORGAN BOUNDARIES Defines a New Family of DNA-Binding Transcription Factors and Can Interact with Specific bHLH Proteins. Nucleic Acids Res..

[174] Uchida N., Townsley B.T., Chung K.-H., Sinha N.R. (2007). Regulation of SHOOT MERISTEMLESS Genes via an Upstream-Conserved Noncoding Sequence Coordinates Leaf Development. Proc. Natl. Acad. Sci. USA.

[175] Xu L., Xu Y.B., Dong A., Sun Y., Pi L., Xu Y., Huang H. (2003). Novel As1 and As2 Defects in Leaf Adaxial-Abaxial Polarity Reveal the Requirement for ASYMMETRIC LEAVES1 and 2 and ERECTA Functions in Specifying Leaf Adaxial Identity. Development.

[176] Horstman A., Bemer M., Boutilier K. (2017). A Transcriptional View on Somatic Embryogenesis. Regeneration.

[177] Tsuwamoto R., Yokoi S., Takahata Y. (2010). Arabidopsis EMBRYOMAKER Encoding an AP2 Domain Transcription Factor Plays a Key Role in Developmental Change from Vegetative to Embryonic Phase. Plant Mol. Biol..

[178] Horstman A., Willemsen V., Boutilier K., Heidstra R. (2014). AINTEGUMENTA-LIKE Proteins: Hubs in a Plethora of Networks. Trends Plant Sci..

[179] Scheres B., Krizek B.A. (2018). Coordination of Growth in Root and Shoot Apices by AIL/PLT Transcription Factors. Curr. Opin. Plant Biol..

[180] Kerstens M., Galinha C., Hofhuis H., Nodine M.D., Scheres B., Willemsen V. (2022). Redundant PLETHORA Activity Promotes Development of Early Embryonic Cell Lineages in Arabidopsis. bioRxiv.

[181] Sauter M. (2015). Phytosulfokine Peptide Signalling. J. Exp. Bot..

[182] Yang H., Matsubayashi Y., Nakamura K., Sakagami Y. (1999). *Oryza sativa* PSK Gene Encodes a Precursor of Phytosulfokine-Alpha, a Sulfated Peptide Growth Factor Found in Plants. Proc. Natl. Acad. Sci. USA.

[183] Igasaki T., Akashi N., Ujino-Ihara T., Matsubayashi Y., Sakagami Y., Shinohara K. (2003). Phytosulfokine Stimulates Somatic Embryogenesis in Cryptomeria Japonica. Plant Cell Physiol..

[184] Kobayashi T., Eun C.-H., Hanai H., Matsubayashi Y., Sakagami Y., Kamada H. (1999). Phytosulphokine-α, a Peptidyl Plant Growth Factor, Stimulates Somatic Embryogenesis in Carrot. J. Exp. Bot..

[185] Umehara M., Ogita S., Sasamoto H., Eun C.-H., Matsubayashi Y., Sakagami Y., Kamada H. (2005). Two Stimulatory Effects of the Peptidyl Growth Factor Phytosulfokine during Somatic Embryogenesis in Japanese Larch (*Larix leptolepis* Gordon). Plant Sci..

[186] Hao Z., Wu H., Zheng R., Li R., Zhu Z., Chen Y., Lu Y., Cheng T., Shi J., Chen J. (2023). A Plant Peptide Hormone Phytosulfokine Promotes Somatic Embryogenesis by Maintaining Redox Homeostasis in *Cunninghamia lanceolata*. Plant J. Cell. Mol. Biol..

[187] Lee H.G., Jang S.Y., Jie E.Y., Choi S.H., Park O.-S., Bae S.H., Kim H.-S., Kim S.W., Hwang G.-S., Seo P.J. (2023). Adenosine Monophosphate Enhances Callus Regeneration Competence for de Novo Plant Organogenesis. Mol. Plant.

[188] Ormancey M., Thuleau P., Combier J.-P., Plaza S. (2023). The Essentials on microRNA-Encoded Peptides from Plants to Animals. Biomolecules.

[189] Guo Y., Qi Y., Yang G., Feng Y., Ding X., Li T., Xue L.-J. (2024). A Genome-Wide Identification of miPEPs in Hybrid Poplar Reveals Regulatory Functions of miPEP166i in Adventitious Root Elongation. Ind. Crop. Prod..

[190] Maher M.F., Nasti R.A., Vollbrecht M., Starker C.G., Clark M., Voytas D.F. (2019). Plant Gene Editing through de Novo Induction of Meristems. Nat. Biotechnol..

[191] Cao X., Xie H., Song M., Lu J., Ma P., Huang B., Wang M., Tian Y., Chen F., Peng J. (2022). Cut–Dip–Budding Delivery System Enables Genetic Modifications in Plants without Tissue Culture. Innovation.

[192] Cao X., Xie H., Song M., Zhao L., Liu H., Li G., Zhu J. (2024). Simple Method for Transformation and Gene Editing in Medicinal Plants. JIPB.

[193] Ding X., Wen C., Yang G., Guo Y., Xue L. (2023). Allele-Specific Transcriptional Regulation of Shoot Regeneration in Hybrid Poplar. Forests.

[194] Guo Y., Feng Y.-F., Yang G.-G., Jia Y., He J., Wu Z.-Y., Liao H.-R., Wei Q.-X., Xue L.-J. (2024). Allele-Specific DNA Methylation and Gene Expression during Shoot Organogenesis in Tissue Culture of Hybrid Poplar. Hortic. Res..

[195] Liao R.-Y., Wang J.-W. (2023). Analysis of Meristems and Plant Regeneration at Single-Cell Resolution. Curr. Opin. Plant Biol..

